# Mapping causal mutations by exome sequencing in a wheat TILLING population: a tall mutant case study

**DOI:** 10.1007/s00438-017-1401-6

**Published:** 2017-11-29

**Authors:** Youngjun Mo, Tyson Howell, Hans Vasquez-Gross, Luis Alejandro de Haro, Jorge Dubcovsky, Stephen Pearce

**Affiliations:** 1 Department of Plant Sciences, University of California, Davis, CA USA; 2 Instituto de Biotecnología, Centro de Investigación en Ciencias Veterinarias y Agronómicas, Instituto Nacional de Tecnología Agropecuaria, Buenos Aires, Argentina; 3 Department of Soil and Crop Sciences, Colorado State University, Fort Collins, CO USA; 4 National Institute of Crop Science, Rural Development Administration, Wanju, South Korea; 5 Howard Hughes Medical Institute, Chevy Chase, MD USA

**Keywords:** Wheat, Exome capture, Mutation mapping, *Rht1*

## Abstract

Forward genetic screens of induced mutant plant populations are powerful tools to identify genes underlying phenotypes of interest. Using traditional techniques, mapping causative mutations from forward screens is a lengthy, multi-step process, requiring the identification of a broad genetic region followed by candidate gene sequencing to characterize the causal variant. Mapping by whole genome sequencing accelerates the identification of causal mutations by simultaneously defining a mapping region and providing information on the induced genetic variants. In wheat, although the availability of a high-quality draft genome assembly facilitates mapping and mutation calling, whole genome resequencing remains prohibitively expensive due to its large genome. In the current study, we used exome sequencing as a complexity reduction strategy to detect mutations associated with a target phenotype. In a segregating wheat EMS population, we identified a clear peak region on chromosome arm 4BS associated with increased plant height. Although none of the significant SNPs seemed causative for the mutant phenotype, they were sufficient to identify a linked ~ 1.9 Mb deletion encompassing nine genes. These genes included *Rht-B1*, which is known to have a strong effect on plant height and is a strong candidate for the observed phenotype. We performed simulation experiments to determine the impacts of sequencing depth and bulk size and discuss the importance of considering each factor when designing mapping-by-sequencing experiments in wheat. This approach can accelerate the identification of candidate causal point mutations or linked deletions underlying important phenotypes.

**Electronic supplementary material:**

The online version of this article (10.1007/s00438-017-1401-6) contains supplementary material, which is available to authorized users.

## Introduction

Forward genetic screens of induced mutant populations have been instrumental in identifying genes responsible for phenotypic variation in plants (Peters et al. 2003). Such screens consist first of isolating a mutant individual exhibiting a phenotype of interest followed by genetic mapping to identify the causal mutation underlying this phenotype. Traditionally, mapping an induced mutation was a lengthy process dependent on identifying genetic markers co-segregating with the target phenotype in a mapping population. A variation of this approach, bulked segregant analysis (BSA) whereby the allelic frequencies of genetic markers are analyzed in two groups of plants exhibiting contrasting phenotypes, can significantly reduce the time and costs of genotyping (Michelmore et al. 1991). Once a mapping interval harboring the causal variant has been defined, candidate genes within this region must be sequenced to identify putative causal mutations underlying the phenotype.

Recent advances in next generation sequencing technologies have reduced sequencing costs to such an extent that for many plant species, it is now feasible to perform whole genome sequencing (WGS) to characterize the full complement of genetic variants within a mutant or a mapping population (Schneeberger 2014). This represents a major advance for genetic mapping studies, since it is possible in a single step to define a mapping interval and identify causal variants associated with the target phenotype (Schneeberger 2014). Mapping-by-sequencing has been widely applied in *Arabidopsis thaliana* to identify causative mutations underlying phenotypes of interest from screens of ethyl methanesulfonate (EMS)-induced mutant populations (Schneeberger et al. 2009; Cuperus et al. 2010; Austin et al. 2011). Typically, mutations are mapped in an F_2_ population derived from a cross between the isolated mutant line and a genetically divergent accession using a BSA-based approach. To identify the causative mutation, high-throughput sequencing is performed on a bulked DNA sample pooled from F_2_ individuals exhibiting the mutant phenotype and the relative frequency of each parental allele within the pool is determined using an informatics package (Schneeberger et al. 2009; Cuperus et al. 2010; Austin et al. 2011). Candidate-induced mutations can subsequently be validated using independent allelic mutants (Schneeberger et al. 2009).

Traditional genetic mapping of induced mutants requires the use of crosses between genetically different individuals to maximize the number of polymorphic genetic markers. By contrast, mapping-by-sequencing characterizes all induced mutations within a population, which can be used as markers, precluding the requirement to cross to a genetically divergent variety. Induced mutations can therefore be mapped using an isogenic F_2_ population developed from a cross between the identified mutant and a non-mutagenized line of the same variety. This reduces both the potentially confounding effect (e.g., epistatic interactions) arising from a heterogeneous genetic background and the number of segregating loci. This approach was originally described in rice (‘MutMap’), whereby WGS was conducted on a bulked DNA sample of individuals from an isogenic F_2_ population which exhibited a mutant phenotype (Abe et al. 2012). Mapping-by-WGS can also be performed without crossing by directly sequencing allelic mutants (Nordström et al. 2013), or sequencing segregating mutant progenies derived from a heterozygous mutant line (Fekih et al. 2013).

To date, mapping-by-sequencing has predominantly been applied in model plant species such as Arabidopsis and rice, which have well-developed genomic resources (Schneeberger et al. 2009; Cuperus et al. 2010; Austin et al. 2011; Abe et al. 2012; Fekih et al. 2013; Nordström et al. 2013). The recent release of high-quality reference genomes and annotated gene models for wheat and barley provides the requisite tools to apply mapping-by-sequencing in these crops (Brenchley et al. 2012; the International Barley Genome Sequencing Consortium 2012; the International Wheat Genome Sequencing Consortium 2014; Chapman et al. 2015; Clavijo et al. 2017). However, despite falling costs, WGS remains prohibitively expensive in wheat and barley due to the high volumes of sequencing data required to provide adequate coverage across their large genomes (the International Barley Genome Sequencing Consortium 2012; the International Wheat Genome Sequencing Consortium 2014). To overcome this limitation, complexity reduction strategies are required. One such approach is exome capture and sequencing, which dramatically reduces sequencing costs, while ensuring coverage of the vast majority of gene coding regions. In barley, a 62 Mb (~ 1.2% of the whole genome) exome capture assay (Mascher et al. 2013) was used to identify variants in protein-coding genes from two phenotypic bulks of an outcrossed F_2_ population, resulting in the identification of an X-ray-induced deletion of the *MANY-NODED DWARF* gene responsible for a shortened plastochron phenotype (Mascher et al. 2014). Similarly, a 110 Mb (~ 0.6% of the whole genome) exome capture assay in hexaploid wheat was used to identify candidate natural variants at the *Yr6* locus responsible for yellow rust resistance from a pooled DNA sample of resistant plants within a doubled haploid population (Gardiner et al. 2016).

Exome sequencing has also been used to facilitate reverse genetic screens by cataloging coding region mutations in EMS-induced TILLING (Targeting Induced Local Lesions IN Genomes) populations in wheat (Krasileva et al. 2017). EMS is an alkylating agent that predominantly induces G-to-A nucleotide substitutions that upon DNA replication also generate C-to-T mutations. A custom-designed 84 Mb capture assay was used to identify over 10 million high-confidence (< 0.2% error rate) EMS-type point mutations in the protein-coding regions of 1535 tetraploid and 1200 hexaploid TILLING lines (Henry et al. 2014; Krasileva et al. 2017). The estimated mutation density of the population was 35–40 mutations per kb, providing, on average, 24 nonsynonymous mutations per annotated wheat gene. This analysis also revealed a high frequency of large deletions (scaffolds with at least five deleted exons) in the hexaploid mutant population. Twenty-nine percent of the 1011 analyzed hexaploid mutant lines (293) showed at least one large deletion, whereas no deletions were detected in the 25 sequenced Cadenza control lines (χ^2^ *P* = 0.0015). This last result suggests that EMS treatment increases the frequency of large deletions in wheat (Krasileva et al. 2017).

In addition to their use in reverse genetics studies, these sequenced mutant populations represent excellent resources for forward genetic screens since the full complement of induced mutations in the protein-coding regions of all lines are known, facilitating precise genotype calling in mapping populations derived from these lines. In the current study, we identified a tall mutant line from this sequenced TILLING population (Krasileva et al. 2017) and describe a mapping strategy to identify the causal induced mutation using exome sequencing in a segregating M_4_ population. Although the approach we describe is designed to identify point mutations, we demonstrate that this method can also identify deletions. In the presented example, while none of the significant SNPs seemed to be causative for the mutant phenotype, they were sufficient to identify a linked deletion encompassing nine genes. These genes include *Rht-B1*, which is a strong candidate for the increased height phenotype. Plant height has a strong effect on crop productivity and an improved understanding of the genes underlying this trait will aid the selection of alternative dwarfing alleles in modern wheat germplasm. The approach we describe can be applied to rapidly identify candidate causative-induced mutations in non-model plant species, including those with large, polyploid genome such as wheat.

## Results

### Tall mutant identification and mapping-by-exome-sequencing

**Fig. 1:**
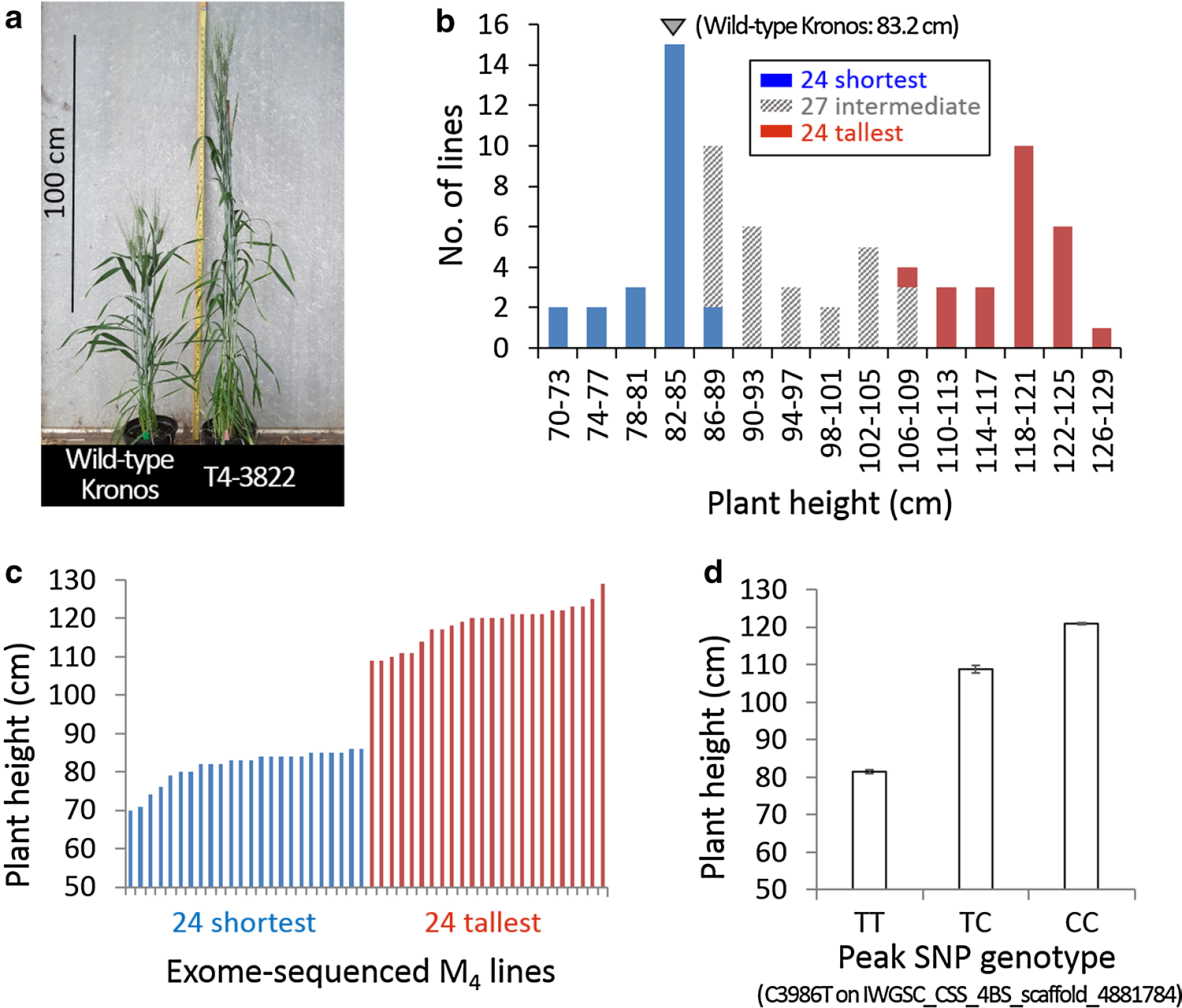
Plant height of non-mutagenized Kronos, the tall mutant T4-3822, and the M_4_ mapping population. **a** Representative plants (six weeks after heading) of non-mutagenized Kronos and a T4-3822 M_4_ sister line carrying the homozygous C allele at the peak SNP (C3986T on IWGSC_CSS_4BS_scaffold_4881784). **b** Plant height distribution in the M_4_ population (*n* = 75). The 24 shortest and 24 tallest lines are highlighted in blue and red, respectively, while the remainder (*n* = 27) of the M_4_ individuals are indicated in gray. A gray triangle denotes the plant height class of non-mutagenized Kronos. **c** Plant height of the 24 shortest (blue) and 24 tallest (red) lines from the M_4_ population selected for exome sequencing. **d** Average plant height ± SE of the M_4_ lines carrying homozygous T allele (*n* = 23), heterozygous C/T (*n* = 8), and homozygous C allele (*n* = 17) at the peak SNP (C3986T on IWGSC_CSS_4BS_scaffold_4881784, Table 1). (Color figure online)

**Fig. 2:**
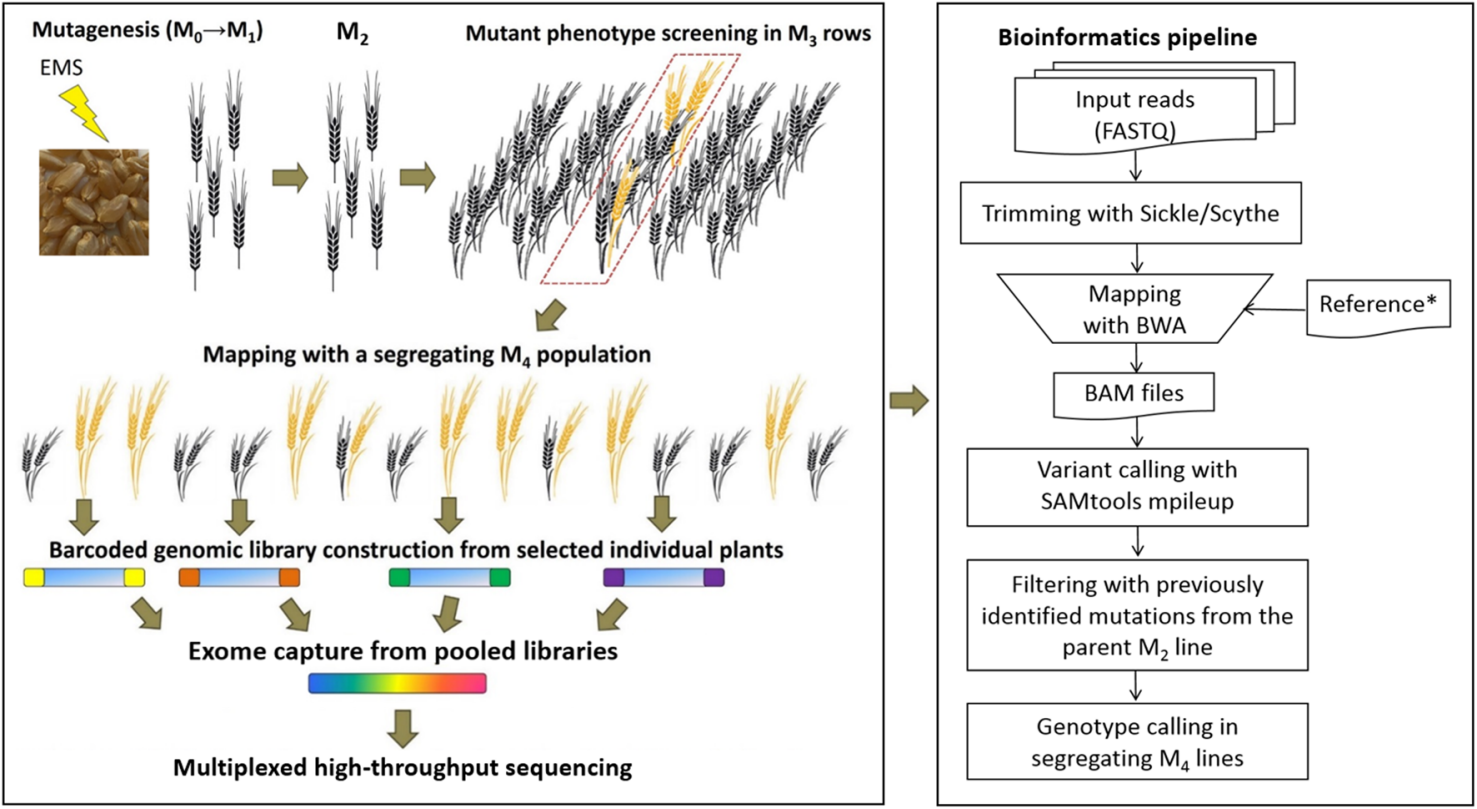
Overview of mutant mapping strategy using exome capture and sequencing. Each M_1_ plant grown from EMS-mutagenized seed was self-pollinated to produce single M_2_ plants, which were exome-sequenced to catalog induced mutations in the protein-coding regions (Krasileva et al. 2017). M_3_ rows derived from each M_2_ plant were screened to identify mutant phenotypes of interest (depicted in yellow). Subsequent M_4_ seeds bulk-harvested from the selected M_3_ row segregating for the mutant phenotype (red dotted box) were used as a mapping population. Barcoded sequencing libraries with unique indices were constructed for each of the selected individual M_4_ plants. Libraries were subjected to exome capture and sequenced in multiplexed reactions. Bioinformatics pipeline for sequencing reads processing, mapping, and genotype calling. *IWGSC CSS reference supplemented with a de novo assembly of unmapped reads from Kronos (Krasileva et al. 2017). (Color figure online)

We performed a visual screen of a tetraploid wheat TILLING population (Uauy et al. 2009) grown as rows in field conditions at the M_3_ generation and identified a mutant line (T4-3822) exhibiting an increased height phenotype when compared to non-mutagenized control lines (Fig. 1a). To study this mutant line further, we bulk-harvested seeds from the M_3_ row and sowed them into field conditions as an M_4_ population (*n* = 75). Within this population, individual plant heights ranged from 70 to 129 cm and exhibited a bimodal pattern of segregation (Fig. 1b). Based on this clear segregating phenotype, we decided to use this material to map the causative mutation using an exome capture and sequencing strategy (Fig. 2). The observed pattern of segregation was consistent with a single causative mutation heterozygous in the M_2_ line. The expected genotype frequency in the M_4_ population of such a mutation is 37.5% (28 lines) homozygous wild-type, 25.0% (19 lines) heterozygous, and 37.5% (28 lines) homozygous mutant. Based on these expected frequencies, we selected the 24 shortest (average height 82 cm) and 24 tallest M_4_ plants (average height 119 cm) for genotyping (Fig. 1c). Exome capture libraries were barcoded with unique indices to retain individual genotype information. Multiplexed sequencing reactions generated, on average, 43.6 million 150 bp reads (21.8 million paired-end reads) per sample (Online Resource Table S1). After trimming for quality and adapter contamination, 97.1% of the processed reads mapped to the reference, similar to the proportion (98%) reported in a previous study using 100 bp paired-end reads (Krasileva et al. 2017). The estimated average sequencing depth across the target exome space [119.2 Mb, (Krasileva et al. 2017)] was 53 × per sample.

**Table 1:** Significant SNPs (Bonferroni adjusted *P* < 0.05) associated with the mutant increased height phenotype

Scaffold^a^		SNP	*P* value	BSA Rank^b^	Effect	Gene		Position^e^	
ID	bp					ID^c^	Description^d^	Chr	Mbp
*IWGSC_CSS_3B_scaff_*						*Traes_3B_*			
10394995	2370	G → A	1.4 × 10^−5^	8	G160S	9FD27C382	Dirigent protein 22-like [*B. distachyon*]	3B	779.1
10351351	3767	G → A	3.3 × 10^−5^	14	Synonymous	0B45CE6A2	n/a	3B	783.5
*IWGSC_CSS_4AL_scaff_*						*Traes_4AL_*			
7173690	939	G → A	6.2 × 10^−6^	54	Intergenic			4A	645.1
7089505	1281	C → T	1.4 × 10^−5^	120	E254K	343B5D587	ATP-binding protein, putative [*M. truncatula*]	4A	674.3
7160166	4006	G → A	1.0 × 10^−5^	18	Intron	590B5CC39	Putative cysteine synthase [*O. sativa*]	4A	675.6
7100865	4872	C → T	2.0 × 10^−5^	42	Synonymous	A710BA481	RNA 2′-phosphotransferase, Tpt1/KptA family [*A. thaliana*]	4A	687.3
7070109	15,757	G → A	3.1 × 10^−6^	n/a	Q187*	71E0C196D	n/a	4A	689.0
7070109	30,790	C → T	1.7 × 10^−5^	n/a	W64*	760AA1408	n/a	4A	689.0
*IWGSC_CSS_4BS_scaff_*						*Traes_4BS_*			
4920499	9117	G → A	5.8 × 10^−6^	12	R74Q	5A606CB40	Type I inositol-1,4,5-trisphosphate 5-phosphatase CVP2 [*A. tauschii*]	4B	12.5
4933555	4037	G → A	8.8 × 10^−7^	23	Synonymous	5AC244927	Endo-1,4-beta-xylanase C [*A. tauschii*]	4B	15.7
1070968	1979	C → T	8.8 × 10^−7^	15	Intergenic			4B	16.7
4872575	19,299	C → T	7.4 × 10^−7^	7	G359R	32B866B33	GDSL esterase/lipase [*A. tauschii*]	4B	20.6
4909391	8166	A → G	3.6 × 10^−6^	5	Intergenic			4B	20.7
4961119	20,843	G → A	1.7 × 10^−6^	4	Intron	F96B8575F	Plant/T23J7-180 protein, putative [*M. truncatula*]	4B	24.5
*4963368*	*3342*	**G** → **A**	*1.6* × *10* ^−*11*^	*3*	*V604M*	*63DD9D036*	*Lipoxygenase 1 [A. tauschii]*	*4B*	*27.2*
*4884290*	*6797*	**C** → **T**	*1.1* × *10* ^−*14*^	*2*	*Intergenic*			*4B*	*27.5*
*4881784*	*3986*	**C** → **T**	< *2.0* × *10* ^−*16*^	*1*	*Synonymous*	*ACBE893D5*	*Phytanoyl-CoA dioxygenase (PhyH) family protein [A. thaliana]*	*4B*	*31.7*
4911525	11,750	C → T	4.8 × 10^−6^	6	5′ UTR	372652643	Maf-like protein [*A. tauschii*]	4B	45.2
4868930	6350	C → T	2.7 × 10^−7^	22	Synonymous	34AF89ACE	Transcription factor bHLH25 [*A. tauschii*]	4B	46.2
2307753	3238	C → T	2.4 × 10^−6^	20	Intron	A53F8B91F	Alpha/beta-Hydrolases superfamily protein [*A. thaliana*]	4B	47.6
4935065	7502	C → T	1.1 × 10^−5^	32	Intron	D4D9B5E3E	Sucrose-phosphate synthase family protein [*A. thaliana*]	4B	93.8
4904809	9422	G → A	3.3 × 10^−5^	39	G133R	BE761A604	Cysteine-rich receptor-like protein kinase 40 [*T. urartu*]	4B	95.3
*IWGSC_CSS_5AL_scaff_*						*Traes_5AL_*			
2796973	5466	G → A	1.6 × 10^−8^	85	3′ UTR	2292EE446	Inositol 1,3,4-trisphosphate 5/6-kinase 4 [*A. thaliana*]	5A	514.2
2808395	209	C → T	3.1 × 10^−7^	107	L35F	EE0639A2E	Flap endonuclease GEN-like protein 1 [*A. tauschii*]	5A	522.1
*IWGSC_CSS_6BL_scaff_*						*Traes_6BL_*			
4217811	4606	C → T	2.8 × 10^−5^	58	V13I	116641644	Calcium-binding EF hand family protein [*A. trichopoda*]	6B	449.8

The three most significant SNPs are in Bold

^a^IWGSC CSS wheat survey sequence version 1

^b^Rank of each SNP ordered by |Δ(SNP-index)| in the BSA simulation (see Fig. 5)

^c^PGSB/MIPS version 2.2

^d^Annotations from orthologues available at Ensembl Plants (http://archive.plants.ensembl.org/index.html). n/a signifies no annotated orthologous gene

^e^IWGSC RefSeq v1.0

In the exome sequencing of the complete Kronos TILLING population, M_2_ line T4-3822 carried 1874 high-confidence EMS-induced SNPs, of which 1247 were heterozygous (Krasileva et al. 2017). We performed genotype calling of each segregating mutation in the 48 M_4_ plants. Using single locus ANOVAs, 25 of these SNPs were significantly associated with height within this population (Bonferroni adjusted *P* < 0.05, Table 1). All these mutations were EMS-type (G-to-A or C-to-T), except for one A-to-G mutation at position 8166 (A8166G) on IWGSC_CSS_4BS_scaff_4909391. Most non-canonical EMS mutations segregating in the TILLING population are due to residual heterogeneity from the parental non-mutagenized seed stock and are present in multiple individuals (Krasileva et al. 2017). However, the A8166G mutation was found only in line T4-3822, and thus is likely to be a genuine induced variant.

Of the 25 significant SNPs, 14 (including the three most significant SNPs) were located on chromosome arm 4BS (12.5–95.3 Mb). The remaining 11 significant SNPs were distributed across four different chromosomes (Table 1). The *P* values of the three SNPs located between 27.2 and 31.7 Mb on chromosome arm 4BS were at least three orders of magnitude lower than the *P* values detected for the SNPs on other chromosomes (Table 1). We considered the region including the three most significant SNPs to be the most likely to harbor the causal mutation. The peak SNP (C3986T on IWGSC_CSS_4BS_scaffold_4881784; *P* < 2.0 × 10^−16^) showed near-perfect co-segregation with the mutant phenotype (Online Resource Table S2). However, this peak SNP could not be the causal mutation because the tall plants (mutant phenotype) were homozygous for the wild-type C allele (SNP in repulsion phase). Among the 48 M_4_ mapping individuals, the 17 plants carrying the wild-type C SNP were on average 49% taller (120.9 cm) than the 23 plants carrying the mutant T SNP (81.4 cm), while eight plants heterozygous for this SNP exhibited intermediate height (108.9 cm, Fig. 1d). This pattern of segregation indicated that the causal mutation was likely to be partially dominant (degree of dominance = 0.39), and tightly linked with the peak SNP.

The second most significant SNP (C6797T on IWGSC_CSS_4BS_scaffold_4884290; *P* = 1.1 × 10^−14^) encoded an intergenic variant (Table 1), and the wild-type allele was associated with the mutant phenotype (Online Resource Table S2). The third most significant SNP (G3342A on IWGSC_CSS_4BS_scaffold_4963368; *P* = 1.6 × 10^−11^) encoded a missense mutation in the coding region of a homolog of *LIPOXYGENASE1* (Table 1). Although the mutant allele was associated with the tall phenotype, this association was much weaker than the peak SNP and three M_4_ plants homozygous for the wild-type allele (GG) at this SNP exhibited a clear increased height phenotype (Table 1; Online Resource Table S2). The other significant SNPs in our analysis had *P* values seven to eleven orders of magnitude greater than the peak SNP (Table 1), and showed weaker association with the increased height phenotype, so we considered them unlikely candidates for the causative mutation (Online Resource Table S2).

**Fig. 3:**
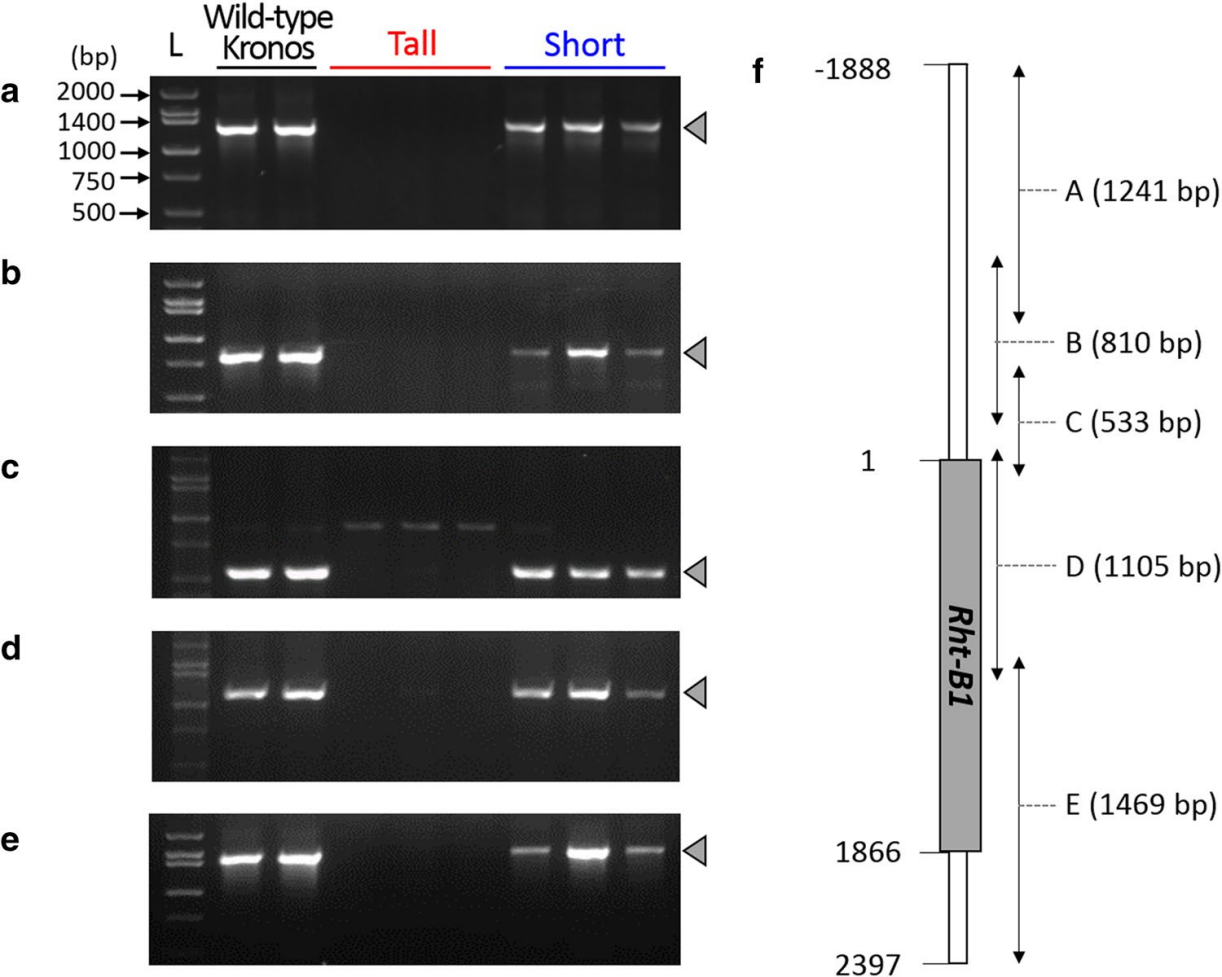
*Rht-B1* deletion in the tall mutant confirmed by PCR. Five PCR reactions were performed in non-mutagenized Kronos and T4-3822 mutants to amplify a 4285 bp region encompassing the complete *Rht-B1* coding region, 1.9 kb of 5′ UTR sequence and 0.5 kb of 3′ UTR. The PCR reactions amplified **a** – 1888 to − 648 bp region. **b** – 986 to − 177 bp region. **c** – 449 to 84 bp region. **d** – 58 to 1047 bp region. **e** 929–2397 bp region. **f** Location of the five primer pairs (Wilhelm et al. 2013b) used in PCRs. The translated region of *Rht-B1* is depicted as a gray box with start and stop codon positions at 1 and 1866 bp, respectively. Target bands in the gel pictures are indicated with gray triangles. *L* ladder (size marker), *Kronos* non-mutagenized Kronos, *Tall* three tallest plants, *Short* three shortest plants from the M_4_ mapping population (Fig. 1b, c). (Color figure online)

Although our initial mutation analysis revealed no obvious candidate point mutations, the highly significant association between the SNPs in the peak region on chromosome arm 4BS and the increased height phenotype suggested that the causal variant was in close proximity. Therefore, we searched for nearby annotated genes and found that *Rht-B1* (30.9 Mb), a gene known to control plant height (Peng et al. 1999), was located 0.8 Mb upstream of the peak SNP (31.7 Mb) and 3.4 and 3.7 Mb downstream of the second (27.5 Mb) and third (27.2 Mb) highest ranked SNPs, respectively. Therefore, we performed PCR to amplify the *Rht-B1* gene and its flanking sequences from tall and short sister lines to search for polymorphisms that might not have been included in the exome capture assay or that were missed by our mutation-calling pipeline. In PCR reactions using a pair of primers specific to *Rht-B1*, we consistently amplified the expected product using template DNA extracted from non-mutagenized Kronos plants and the three shortest sister lines, but all amplifications failed in the three tallest sister lines (Fig. 3a). We then performed PCRs with four additional primer pairs to amplify an overlapping region totaling 4285 bp, encompassing the complete *Rht-B1* coding region, including the 5′ and 3′ UTRs (Wilhelm et al. 2013b) and found consistent results (Fig. 3b–e). PCR amplifications of *Rht-B1* from the 48 M_4_ lines also failed in all 17 tall lines homozygous for the C allele at the peak marker (C3986T on IWGSC_CSS_4BS_scaffold_4881784), while 23 short lines homozygous for the T allele and eight heterozygotes produced expected amplicons (Online Resource Fig. S1). This result demonstrates that the increased height phenotype in this population is associated with the complete deletion of a chromosome region including *Rht-B1*.

### Coverage analysis to define the deletion size

**Fig. 4:**
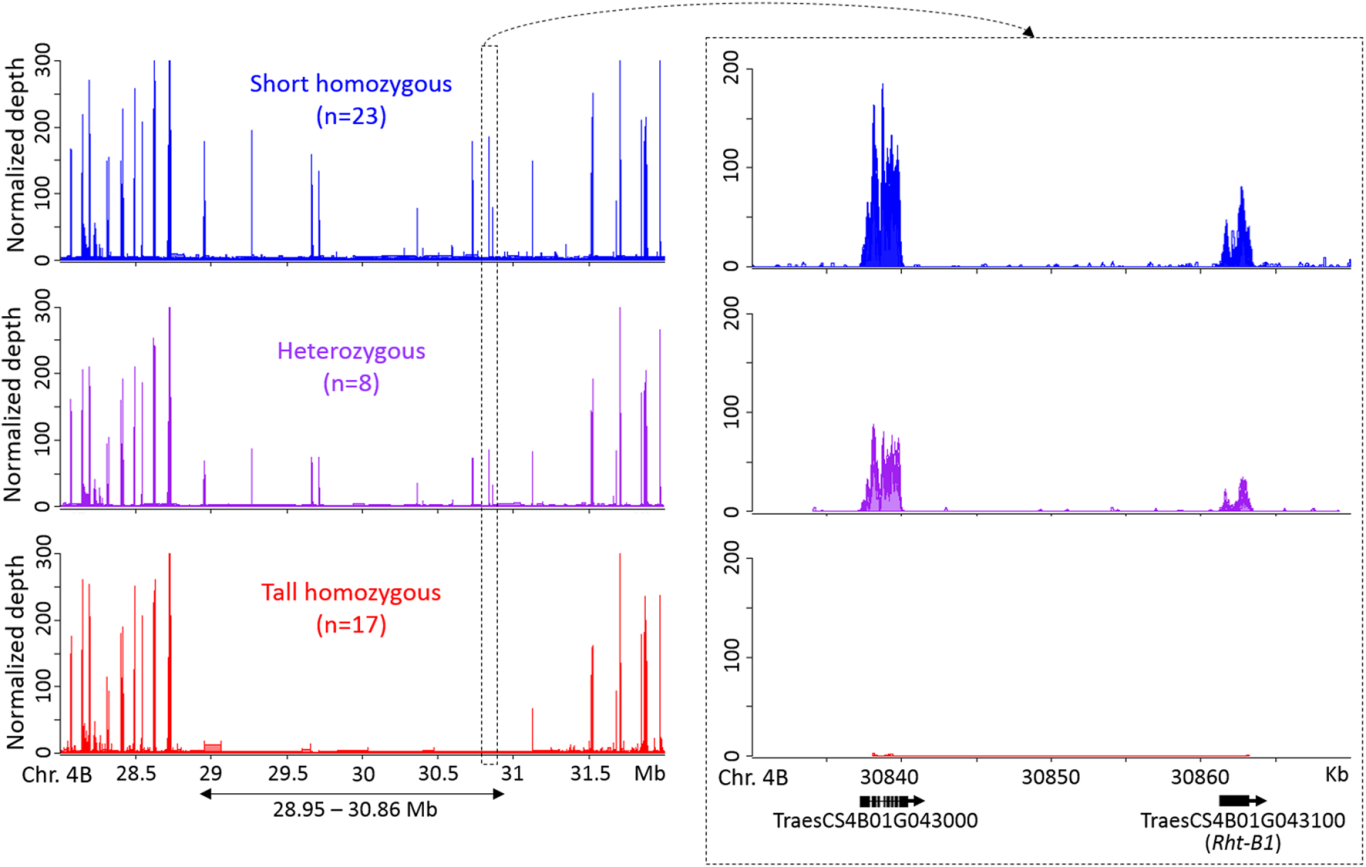
Coverage analysis of chromosome 4B near the peak region. Normalized read depth of the M_4_ mapping individuals in the 28–32 Mb region on chromosome 4B. The double-headed arrow below the graph indicates the ~ 1.9 Mb deletion identified in the tall individuals carrying the homozygous C allele at the peak SNP (C3986T on IWGSC_CSS_4BS_scaff_4881784). The box with the dotted line indicates detailed coverage of the 40 kb region (30.83–30.87 Mb) harboring *Rht-B1* (TraesCS4B01G043100) and its neighboring gene (TraesCS4B01G043000). Gene models are illustrated at the bottom of the graph, with arrows indicating gene orientation. Blue, purple, and red colors correspond to M_4_ individuals homozygous (CC), heterozygous (CT) or homozygous (TT) for the peak SNP (C3986T on IWGSC_CSS_4BS_scaff_4881784) (Online Resource Table S2). (Color figure online)

To define the size of the deletion, we conducted coverage analysis of the region of chromosome 4B surrounding *Rht-B1* by re-mapping the exome sequencing reads from the 48 M_4_ mapping individuals to the IWGSC RefSeq v1.0 wheat genome assembly. To eliminate complications from heterozygotes, coverage analysis of the eight M_4_ lines heterozygous for the peak SNP (C3986T on IWGSC_CSS_4BS_scaffold_4881784) was conducted separately from the 23 short and 17 tall M_4_ sister lines homozygous for this SNP. This analysis revealed a region on chromosome 4B (28.95–30.86 Mb) encompassing nine annotated genes which all showed high coverage for the 23 homozygous short sister lines, but almost zero coverage in the 17 homozygous tall sister lines (Fig. 4; Table 2). The one exception was TraesCS4B01G042800, which was not included in the exome capture design and thus exhibited low coverage in all M_4_ lines (Online Resource Fig. S2). Normalized read depth from the eight heterozygous lines in this region was approximately half that of the 23 homozygous short sister lines, indicating hemizygosity (Fig. 4; Online Resource Fig. S2). The genes immediately upstream and downstream of the deleted region exhibited similar levels of coverage among all 48 lines (Fig. 4; Online Resource Fig. S2). Taken together, this analysis reveals that the tall mutant sister lines from this population carry a ~ 1.9 Mb deletion on chromosome 4B encompassing nine genes, including *Rht-B1*.

**Table 2:** Nine annotated genes within the 28.95–30.86 Mb deletion on chromosome 4B in the T4-3822 tall mutant

Gene ID^a^	Description^b^	Location
TraesCS4B01G042300	Oxysterol-binding protein-related protein 1C isoform X2 [*A. tauschii*]	chr4B:28949569–28960040
TraesCS4B01G042400	Phosphatidylinositol 4-phosphate 5-kinase 1-like [*A. tauschii*]	chr4B:29267113–29270732
TraesCS4B01G042500	Protein FAF-like, chloroplastic [*A. tauschii*]	chr4B:29673211–29674674
TraesCS4B01G042600	Ruvbl1 protein-like [*O. sativa*]	chr4B:29710372–29715009
TraesCS4B01G042700	Teosinte branched 1 protein [*T. aestivum*]	chr4B:30362277–30363341
TraesCS4B01G042800	Unnamed protein product [*T. aestivum*]	chr4B:30498971–30504757
TraesCS4B01G042900	Zinc finger C3HC4 type domain-containing protein [*T. aestivum*]	chr4B:30725178–30734116
TraesCS4B01G043000	EamA domain-containing protein [*T. aestivum*]	chr4B:30837210–30840295
TraesCS4B01G043100	RHT-B1 protein [*T. aestivum*]	chr4B:30861382–30863247

^a^IWGSC RefSeq v1.0 annotation

^b^Description obtained from ‘BLASTx’ search (“nr” database; default parameters) using the gene sequences as queries

### BSA simulation

We identified the putative causative variant in this mutant line using individually barcoded sequencing libraries from 48 mapping individuals. To determine whether a bulked sequencing approach using DNA samples from two contrasting phenotypic groups would also have been effective, we performed a simulated pooling experiment. Sequencing reads from the 48 M_4_ individuals were divided into two bulks based on their phenotype, i.e., the 24 shortest lines as a wild-type bulk (W-bulk) and the 24 tallest lines as a mutant bulk (M-bulk, Fig. 1b, c). The average estimated sequencing depth across the 119.2 Mb target exome region was 53 × per line, resulting in an estimated sequencing depth per bulk of 1,272 × in our BSA simulation (53 × multiplied by 24 lines). It is important to note that the level of coverage used for this simulation is significantly higher than the recommended coverage for a BSA mapping experiment. We calculated the mutant allele frequency for each of the segregating SNPs [SNP-index (Abe et al. 2012)] within each bulk. The expected SNP-index for a segregating mutation not linked to the causative mutation is 0.5 in both W-bulk and M-bulk. For such mutations, the |Δ(SNP-index)| value (=|SNP-index (M-bulk)—SNP-index (W-bulk)|) is expected to be close to zero (Fekih et al. 2013). By contrast, mutations tightly linked to the causative mutation would be expected to have |Δ(SNP-index)| values close to one.

**Fig. 5:**
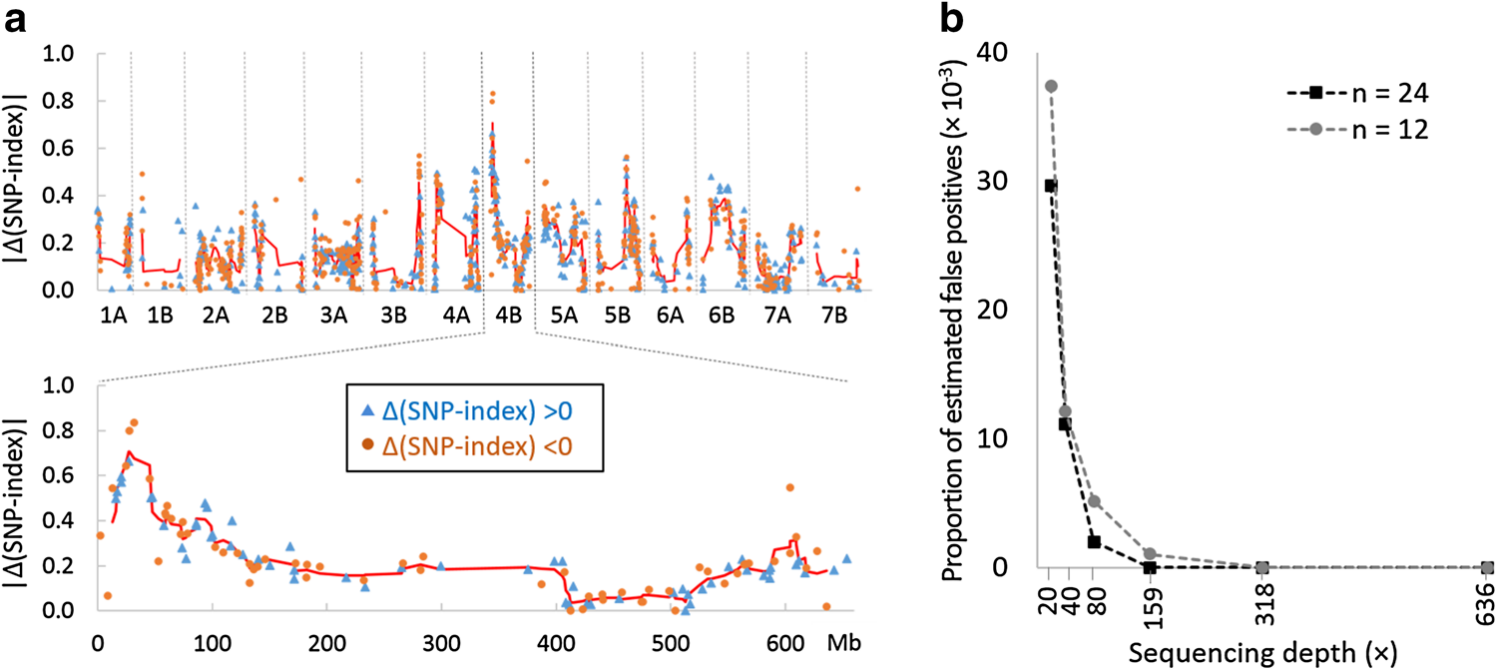
BSA and subsampling simulations under different sequencing depths and bulk sizes. **a** |Δ(SNP-index)| at each SNP position across the 14 tetraploid wheat chromosomes, with a detailed plot of chromosome 4B (sequencing depth = 1320×, bulk size = 24). Δ(SNP-index) at each position is calculated by subtracting the SNP-index (mutant allele frequency) in the W-bulk (24 shortest M_4_ lines) from that in the M-bulk (24 tallest M_4_ lines) [= SNP-index (M-bulk) − SNP-index (W-bulk)]. Blue triangles indicate SNPs with higher frequencies in the mutant bulk [SNP-index (M-bulk) > SNP-index (W-bulk)], while orange circles indicate SNPs with higher frequencies in the W-bulk [SNP-index (W-bulk) > SNP-index (M-bulk)]. Sliding window average (red) is plotted by averaging the |Δ(SNP-index)| values of five consecutive SNPs and shifting the window by one SNP at a time. **b** Proportion of false positives estimated by counting the number of SNPs with |Δ(SNP-index)| ≥0.8 outside the peak candidate region (chromosome 4B, 12.5–95.3 Mb; Table 1, Online Resource Text S1). (Color figure online)

We plotted the absolute value of Δ(SNP-index) for each segregating SNP to identify chromosomal regions exhibiting the highest |Δ(SNP-index)| values (Fig. 5a). Consistent with our original analysis, this approach revealed the distal region of chromosome arm 4BS as the top candidate (Fig. 5a; Table 1): seven SNPs located at 20.6–45.2 Mb on chromosome arm 4BS had the highest |Δ(SNP-index)| values, ranging from 0.57 to 0.83. This result shows that a BSA-based approach gave consistent results with our ANOVAs based on individual barcoded libraries (Table 1), identifying the same three SNPs as the most significant variants.

We next studied the effect of bulk size on mapping accuracy by performing a series of simulations using bulk sizes ranging from 1 to 24 individuals (Online Resource Fig. S3). The three most significant SNPs (C3986T on IWGSC_CSS_4BS_scaff_4881784, C6797T on IWGSC_CSS_4BS_scaff_4884290, and G3342A on IWGSC_CSS_4BS_scaff_4963368) were consistently ranked in the same order until bulk size decreased to 11, indicating that in our study, the same peak region could be mapped using smaller bulk sizes (Online Resource Fig. S3a). Furthermore, the two most significant SNPs were consistently ranked even in bulk sizes of 5, although in smaller bulk sizes, high |Δ(SNP-index)| values of several unlinked SNPs meant that this ranking was lost (Online Resource Fig. S3a). The |Δ(SNP-index)| values of the top three SNPs increased as bulk size decreased from 24 to 13 (Online Resource Fig. S3b). This was likely due to enhanced phenotype accuracy obtained through selecting mapping individuals with more extreme phenotypes, which removed heterozygous lines that had been included in the larger bulks (Online Resource Table S2).

We next performed another simulation to study the effect of sequencing depth on BSA mapping at two fixed bulk sizes (*n* = 24 and *n* = 12) (Fig. 5b; Online Resource Fig. S4). In both bulk sizes, noise from false positives (defined as the proportion of SNPs with |Δ(SNP-index)| ≥0.8 outside the peak candidate region, chromosome 4B, 12.5–95.3 Mb) began to increase markedly as sequencing depth per bulk decreased below 80× (Fig. 5b; Online Resource Fig. S4). However, even with an increasing rate of false positives under decreasing sequencing depth, a sliding window approach averaging the |Δ(SNP-index)| values of five consecutive SNPs identified the same SNP (C3986T on IWGSC_CSS_4BS_scaffold_4881784) as the most significant even at 20×, the lowest simulated sequencing depth (Online Resource Fig. S4).

## Discussion

### Mapping-by-sequencing in species with complex genomes

Mapping-by-sequencing is a powerful approach to identify genes responsible for phenotypes of interest from forward genetic screens. The availability of high-quality genome assemblies for most crop plants, including wheat, will facilitate its application in studies of agronomic traits (Brenchley et al. 2012; the International Wheat Genome Sequencing Consortium 2014; Chapman et al. 2015; Clavijo et al. 2017). However, whole genome resequencing, the favored approach for mapping induced mutations in model plant species, remains prohibitively expensive in wheat due to its large genome size, necessitating a complexity reduction strategy. In the current study, we used an exome capture assay equivalent to approximately 1% of the wheat genome (Krasileva et al. 2017). Focusing on the exome dramatically reduces sequencing costs while ensuring coverage of all predicted protein-coding genes included in the assay. Repetitive sequences, which account for more than 85% of the wheat genome, are excluded.

One obvious drawback of exome sequencing is that causal mutations in non-coding regions, such as promoters or large introns outside the captured sequence (> 300 bp from exon borders), will go undetected. An analysis of open chromatin regions of the maize genome revealed regulatory regions totaling < 1% of all sequences that were responsible for approximately 40% of observed phenotypic variation (Rodgers-Melnick et al. 2016). In genetic mapping experiments, capture assays including both exomes and regulatory regions of the genome will greatly increase the chances of identifying different types of causal mutations. In wheat, regulatory capture assays that include promoters and non-overlapping open chromatin regions will capture roughly similar genome regions as those obtained by exome capture [~ 120 Mb in tetraploid wheat and ~ 180 Mb in hexaploid wheat (Krasileva et al. 2017)] while doubling the sequencing costs. With decreasing costs, the sequencing of < 400 Mb will still be a reasonable investment to identify a causal mutation.

Even when a causative mutation is not sequenced using currently available exome capture assays, mapping information from nearby SNPs will likely be sufficient to identify a genomic interval containing the causal mutation. In such cases, subsequent characterization and sequencing experiments of the mapped region will be required, as shown in the current study. Although we did not directly identify the causative mutation, the location information provided by our SNP analysis was sufficient to identify the linked deletion associated with the mutant phenotype.

One advantage of working with relatively recently formed polyploid species is their tolerance of high levels of mutations (average 2705 and 5351 mutations per individual in the exomes of tetraploid and hexaploid wheat, respectively (Krasileva et al. 2017)), providing a large number of markers to delimit the candidate region in populations generated from crosses between induced mutants and the original non-mutagenized line.

In addition to exome capture, other complexity reduction strategies have been applied to map mutations in crop species. In maize, natural and induced causal variants in *glossy3* and *glossy13* genes were mapped using markers derived from RNAseq data in an approach known as Bulked Segregant RNA-seq (BSR-Seq) (Liu et al. 2012; Trick et al. 2012; Li et al. 2013; Ramirez-Gonzalez et al. 2015). In this approach, sequencing costs are similarly reduced by focusing on the expressed portion of the genome and expression data can aid subsequent candidate gene validation and characterization. However, BSR-seq also has limitations because it is only possible to detect variants that fall within genes expressed in the tissues and developmental stages/environments selected for the experiment.

Alternatively, when prior knowledge of the nature of the mutation and multiple independent mutant alleles are available, highly targeted enrichment strategies can be used. One example is R-gene enrichment sequencing (‘MutRenSeq’), whereby a capture assay including only genes that encode nucleotide binding and leucine-rich repeat (NLRs) proteins was used to clone two stem rust resistance genes, *Sr22* and *Sr45*, from an EMS-induced mutant population (Steuernagel et al. 2016). In a separate study, the *Pm2* gene for powdery mildew resistance was cloned in hexaploid wheat by sequencing a single flow-sorted chromosome known to harbor the resistant locus (‘MutChromSeq’) (Sánchez-Martín et al. 2016). For most general applications in wheat where such *a priori* knowledge is not available, mapping-by-exome-sequencing is likely to be cost-effective. However, if the current trend of falling sequencing costs continues, whole genome resequencing may soon become feasible even for large genome species such as wheat.

### Factors to consider in mapping-by-sequencing experiments

In addition to complexity reduction, a number of other factors must be considered when designing a mapping-by-sequencing experiment. We briefly discuss each of these factors in the sections below.

### Population type

In the current study, we mapped the causative mutation directly in an M_4_ population derived from an EMS-mutagenized tetraploid M_2_ wheat line that had previously been exome-sequenced (Krasileva et al. 2017). Prior knowledge of the high-confidence segregating mutations facilitated genotyping in the mapping population. However, such prior information is not a prerequisite for mapping-by-sequencing, since sequencing variants can be directly identified in the segregating population. We chose to map the causative mutation directly in an M_4_ population since we observed a clear segregating phenotype, allowing us to bypass the two generations required to generate a cross and develop an F_2_ mapping population.

### BSA vs individual libraries

The use of individually barcoded exome capture libraries allowed us to retain genotype information of each mapping individual. This information accelerated the identification of heterozygous individuals for the candidate region, which helped us to re-analyze the data without the potentially confounding effects of heterozygotes (Fig. 4; Online Resource Fig. S2). This individual haplotype information was also useful to identify recombination break points and to accelerate the identification of potential candidate genes. In the current study, this information allowed us to define the borders of the deletion without generating additional markers. However, the benefits of this additional information are counterbalanced by the higher experimental costs associated with barcoding, and constructing multiple individual libraries and exome capture assays. Our simulation of a BSA experiment using the exome-sequencing data showed that even with imperfect phenotyping (i.e., inclusion of eight heterozygotes in bulk samples), the BSA analysis identified the same closest SNPs when appropriate coverage was used (Fig. 5; Online Resource Fig. S3). This observation suggests that for either qualitative traits or quantitative traits exhibiting clearly separated phenotypes (as the one tested in this study) BSA would likely be a more cost-efficient mapping approach.

### Population and bulk sizes

In the current study, we mapped the causative mutation using a relatively small population size (*n* = 75), selecting the 24 shortest and 24 tallest lines for genotyping based on expected allele frequencies. The optimal population size will depend on the heritability of the trait (phenotyping accuracy) and will likely be larger for traits with lower heritability. Larger segregating populations facilitate the selection of larger and more homogenous pools, improving mapping resolution.

Larger bulk sizes are expected to improve mapping resolution by increasing the chance of having more recombinants near the causal variant (James et al. 2013), but will also increase the chances of including incorrectly phenotyped individuals. In our study, decreasing bulk size from 24 to 13 individuals from the extremes of the distribution in each of the bulks consistently identified the same top three SNPs as the most likely candidates (Online Resource Fig. S3). Furthermore, mapping results were clearer in smaller bulks and gave greater |Δ(SNP-index)| values at the peak region than when using a bulk size of 24 (Online Resource Figs S3 and S4), because heterozygous individuals were removed in smaller bulks (Online Resource Table S2). Therefore, based on the current study, a bulk size of 11–13 individuals seems optimal.

### Sequencing depth

Recommendations of sequencing depth per bulk when conducting mapping-by-WGS ranges from 10× to 40× (Schneeberger et al. 2009; Abe et al. 2012; James et al. 2013; Garcia et al. 2016). However, as exome capture efficiency is variable in different regions of the genome, sequencing coverage is also variable, and a large number of regions with poor coverage are found even under relatively high (> 20×) average sequencing depth (Krasileva et al. 2017). We simulated the effect of reduced sequencing coverage and found that noise from false positives began to increase markedly when the average sequencing depth per bulk dropped below 80× (Fig. 5b, c). Therefore, our results suggest that sequencing coverage of at least 80 × should be targeted when conducting mapping-by-exome-sequencing.

### The deletion of *Rht-B1b* is likely responsible for increased height phenotype

Although none of the significant SNPs mapping to the peak region on chromosome arm 4BS were causative for the tall phenotype (Table 1), the positional information from this mapping allowed us to determine that the tall mutants carried a ~ 1.9 Mb deletion completely linked to the peak SNP. In addition to the high density of EMS-induced point mutations in TILLING populations, large deletions of more than five adjacent exons are also present at a higher frequency in EMS treated than in non-mutagenized control plants (Krasileva et al. 2017). However, since the natural rate of deletions in polyploid wheat is relatively high (Dvorak et al. 2004; Dvorak and Akhunov 2005), it is not possible to determine if the identified deletion was caused by EMS or by an EMS-independent mechanism. Our capture assay does not include repetitive regions so we could not precisely establish the borders of the deleted region. However, we were able to determine that this deletion occurs in a chromosomal region that is highly conserved among several *Poaceae* species (Duan et al. 2012; Wilhelm et al. 2013a) and harbors at least nine annotated genes including wheat orthologs of *TEOSINTE BRANCHED1* (*TB1*, TraesCS4B01G042700), a C3HC4-Type RING Finger (TraesCS4B01G042900) and *Rht-B1* (TraesCS4B01G043100, Table 2). To identify which of these genes is causative for the increased height phenotype, it will be necessary to characterize independent knockout mutants for each of the deleted genes. This process will be facilitated by the in silico TILLING databases (Krasileva et al. 2017). A screen of this database revealed that for seven of the nine genes, individuals carrying mutations that introduce premature stop codons or that disrupt splice sites are available (Online Resource Table S4). Among these mutants, we identified one tetraploid line carrying a mutation encoding a premature stop codon in *Rht-B1* (W424* in line T4-3545, Online Resource Table S4). Plants homozygous for this mutation are significantly taller than wild-type plants, while individuals heterozygous for the mutation are intermediate in height (Online Resource Fig. S5). This suggests that the deletion of *Rht-B1* is the most likely explanation for the results observed in the mutant reported in this study. The wheat variety Kronos carries the gain-of-function *Rht-B1b* allele that encodes a constitutively active repressor of gibberellin (GA) signaling which confers a semi-dwarf phenotype (Peng et al. 1999), and therefore, its deletion is expected to generate taller plants. In a large suppressor screen for tall mutants in a wheat variety carrying the dwarf *Rht-B1c* allele, more than 150 independent tall mutant lines carried deletions of various sizes that all included *Rht-B1* (Chandler and Harding 2013; Miraghazadeh et al. 2016). In addition, the T4-3822 mutant showed restored GA sensitivity at the seedling stage (Online Resource Fig. S6), providing additional indirect evidence that the deletion of *Rht-B1* in T4-3822 is causative for the increased height phenotype. However, since there is evidence linking the deleted *TB1* and members of the C3HC4-Type RING Finger family to differences in plant height in other species (Lewis et al. 2008; Wu et al. 2014), we cannot rule out their contribution to the differences in plant height.

## Conclusions

Using exome sequencing in a small segregating M_4_-induced mutant population, we identified a 1.9 Mb deletion responsible for an increased height phenotype in wheat. The use of individually barcoded exome capture libraries was more expensive than BSA, but provided additional information that accelerated the delimitation of the region encompassing the causal mutation. The approach we describe here can be applied to map causative mutations underlying agriculturally important traits in non-model species, including those with large, repetitive genomes.

## Materials and methods

### Plant material

A tetraploid wheat TILLING population (variety ‘Kronos’) was previously developed using EMS (Uauy et al. 2009). Part of this population (733 M_3_ lines, each derived from a unique M_2_ individual plant) was grown in one-meter head rows, with 30 seeds per line, in the field at the University of California, Davis, CA (38° 22′ N, 121° 46′ W) in the 2013–14 growing season. Through a visual screen, we identified a mutant line (T4-3822) which exhibited an increased height phenotype segregating within the M_3_ row. M_4_ seeds were bulk-harvested from the M_3_ row and sown in the same field location in the 2014–15 growing season. Seventy-five M_4_ seeds were sown in 15 one-meter rows to maintain five individual plants per row. We measured final height at maturity and selected the 24 shortest and 24 tallest plants for genotyping by exome sequencing.

### Genomic library preparation, exome capture and sequencing

Genomic DNA was extracted from seedling leaf tissue of the selected M_4_ plants using the CTAB (cetyltrimethylammonium bromide) method (Murray and Thomson 1980). Fragmentation, genomic library construction and exome capture were conducted as described previously (Krasileva et al. 2017). Briefly, genomic DNA normalized at 200 ng/µl was sheared using the E220 Focused-ultrasonicator (Covaris, Woburn, MA, USA) to produce an average fragment size of 350 bp. Genomic libraries were prepared using the KAPA HTP Library Preparation Kit (Kapa Biosystems, Wilmington, MA, USA) and each library was indexed using a unique NEXTflex-96™ DNA Barcode (Bioo Scientific, Austin, TX, USA) to allow multiplexed exome capture and sequencing reactions. Library preparation procedures including end repair, poly-A tailing, and adapter ligation were performed using the Sciclone G3 Liquid Handling Workstation (PerkinElmer, Norwalk, CT, USA) at the UC Davis Genome Center. Libraries were quantified with a Qubit^®^ 2.0 Fluorometer (Thermo Fisher Scientific, Waltham, MA, USA), amplified by eight cycles of PCR following the KAPA HTP Library Preparation Kit protocol (KR0426-v4.15; Kapa Biosystems, Wilmington, MA, USA), and purified with the Agencourt AMpure XP system (Beckman Coulter, Fullerton, CA, USA). Eight amplified libraries were pooled (150 ng each) per capture reaction and six captures were prepared using the 84 Mb SeqCap EZ Design (140228_Wheat_Dubcovsky_D18_REZ_HX1 for *T. turgidum*; Roche Nimblegen, Madison, WI, USA), which targets 286,799 exons (219,383 of which were padded with 30 bp of intronic sequence on either side) from 82,511 non-redundant wheat transcripts (Krasileva et al. 2017). Although the assay design covers 84 Mb of gene space, capture coverage averages 119.2 Mb in tetraploid wheat due to the capture of homologous sequences not included in the assay design (Krasileva et al. 2017). Exome libraries were sequenced on the HiSeq 3000 platform (Illumina, San Diego, CA, USA) using the paired-end 150 bp (PE 150) module at the UC Davis Genome Center.

### Bioinformatics and statistical analysis

Raw sequencing reads were processed using ‘Sickle’ (version 1. 33; default parameters with -l 20, https://github.com/najoshi/sickle) and ‘Scythe’ (version 0. 991; default parameters except -p 0.4, https://github.com/vsbuffalo/scythe) to remove low quality and contaminating adapter sequences, respectively. Using ‘BWA’ (version 0. 7. 9a; command “aln” with default parameters; command “sampe” with default parameters except -n 10) (Li and Durbin 2009), the processed reads were mapped to a custom reference sequence comprising the International Wheat Genome Sequencing Consortium (IWGSC) Chromosome Survey Sequences (CSS) supplemented with a de novo assembly of unmapped reads from Kronos (Krasileva et al. 2017). Sequence Alignment/Map (SAM) files for the 48 samples were generated, converted to the Binary Alignment/Map (BAM) format and sorted with ‘SAMtools’ (version 1.3.1) (Li et al. 2009). Variant calling was conducted using ‘SAMtools’ (command “mpileup” with default parameters except -d 8000, -Q 20, and -q 20). Using a custom script, each M_4_ line was genotyped for the 1874 EMS-induced mutations which had previously been characterized in the coding regions of the M_2_ tall mutant line (Krasileva et al. 2017) using the following thresholds: sequencing depth ≥ 5 and allele calling frequency ranges of > 90% for homozygous and 30–70% for heterozygous alleles. After genotype calling, single locus analysis of variance (ANOVA) was conducted on plant height using R (version 3.3.1) to identify mutations significantly associated with the phenotype in the 48 M_4_ individuals.

### Polymerase chain reaction (PCR)

To confirm the deletion of the complete *Rht-B1* gene in Kronos tall mutant line T4-3822, we used five pairs of homeolog-specific PCR primers described previously [Online Resource Table S3, (Wilhelm et al. 2013b)] to amplify overlapping DNA fragments encompassing the *Rht-B1* coding region (1866 bp), 1.9 kb of 5′ UTR sequence and 0.5 kb of 3′ UTR sequence. PCRs were performed using template DNA from non-mutagenized Kronos, the three shortest and three tallest individuals from the M_4_ mapping population using PCR conditions previously reported (Wilhelm et al. 2013b). Each PCR was conducted in a 20 µL reaction volume containing 100 ng template DNA, 0.25 µM of forward and reverse primers, 0.1 mM of each dNTP, 0.8 µL *Taq* polymerase, 1.5 mM MgCl_2_, 5% DMSO, and 10 × PCR buffer. Reaction conditions consisted of an initial denaturation at 94 °C (5 min), 40 cycles of 94 °C (20 s), 60 °C (30 s), and 72 °C (1 min), followed by the final extension at 72 °C (7 min). PCR products were visualized on a 1.5% agarose gel using ethidium bromide.

### GA sensitivity assay

GA sensitivity assays were conducted using wild-type seedlings of the varieties Kronos (*Rht-B1b*, GA insensitive) and Gredho (*Rht-B1a*, GA sensitive), and a T4-3822 M_5_ line homozygous for the deletion on chromosome arm 4BS. Seeds were sown 2.5 cm below the top edge of germination paper (26 × 13 cm^2^) moistened with distilled water, kept at 4 °C for 48 h, and moved to room temperature in solutions containing different concentrations (0, 0.1, 1, and 10 µM; dissolved in distilled water) of GA_3_ (Sigma-Aldrich, St. Louis, MO, USA). Coleoptile and shoot length were measured after 10 days. The experiment was conducted as a randomized complete block design with four blocks, one replication (eight subsamples) per block/treatment combination.

### Coverage analysis

To define the size of the identified deletion on chromosome 4B, we conducted a sequencing coverage analysis. The processed sequencing reads from the 48 M_4_ individuals were re-mapped to the IWGSC Reference Sequence (RefSeq) v1.0 assembly (https://wheat-urgi.versailles.inra.fr/Seq-Repository/Assemblies) using ‘BWA’, and BAM files were generated as described above. To minimize confounding effects from reads with multiple mapping positions, we extracted only reads with Phred-scaled mapping quality score > 20 using ‘SAMtools’ (command “view” with -q 20), which excludes reads mapping to multiple locations. ‘BEDGRAPH’ files for chromosome 4B were created using ‘Bedtools’ (version 2.25; command “genomecov” with default parameters) (Quinlan and Hall 2010). Read depth of each sample was normalized to the average sequencing depth, and visualized using the R/Bioconductor package ‘Sushi’ (Phanstiel et al. 2014).

### BSA simulation

To compare the effectiveness of sequencing individual libraries versus a BSA approach to identify causative variants, we simulated sequencing two different pools of DNA samples using the same genotyping data: one from the 24 tallest M_4_ lines and the other from the 24 shortest M_4_ lines. Read numbers of the 24 individuals belonging to each phenotypic group were summed at each of the 1874 SNPs and the SNP-index (Abe et al. 2012) in each bulk was calculated by dividing the number of reads for a mutant allele by the number of total reads at each SNP position. To eliminate low quality SNPs or regions with strong segregation distortion, we eliminated those SNPs which were present at very high frequency (> 0.8) in both bulks or at very low frequency (< 0.2) in both bulks. We then calculated a Δ(SNP-index) (Abe et al. 2012; Fekih et al. 2013) by subtracting the SNP-index of the wild-type bulk from that of the mutant bulk at each SNP and plotted its absolute value (|Δ(SNP-index)|) at each SNP position according to the IWGSC RefSeq v1.0 assembly. This was determined by ‘BLASTn’ (version 2.2.29; default parameters) searches with the IWGSC CSS scaffolds sequences containing each SNP as queries. Following the sliding window approach described in Abe et al. (2012), the average |Δ(SNP-index)| values of five consecutive SNPs were plotted by shifting the window by one SNP at a time.

We also conducted simulations to test the impact of bulk size and sequencing depth on BSA results. Bulk size simulation was performed by decreasing bulk size from *n* = 24 (24 shortest and 24 tallest lines in each bulk) through *n* = 1 (one shortest and one tallest line in each bulk) using a custom Python script (https://github.com/DubcovskyLab/mapping_by_exome_seq_Mo_et_al). Sequencing depth simulation was performed by randomly subsampling different proportions of mapped reads (50, 25, 12.5, 6.25, 3.13, and 1.56%) using ‘SAMtools’ (view; -s followed by the desired proportion). Subsampled BAM files were analyzed using the same informatic tools and parameters described above to generate genotype data of the 48 mapping individuals. BSA simulation was conducted by calculating |Δ(SNP-index)| as described above, with two different bulk sizes (*n* = 24 and *n* = 12). The number of false positives was estimated at each sequencing depth under the two bulk sizes by counting the number of SNPs with |Δ(SNP-index)| ≥0.8 outside the peak candidate region (chromosome 4B, 12.5–95.3 Mb). Further details on the calculations used to estimate the proportion of false positives are provided in Online Resource Text S1.

## Electronic supplementary material

Below is the link to the electronic supplementary material.

Supplementary material 1 (PDF 1724 KB)
