# Supplementary material for: Mapping causal mutations by exome sequencing in a wheat TILLING population: a tall mutant case study
Source: Mol Genet Genomics. 2017 Nov 29;293(2):463–77. doi: 10.1007/s00438-017-1401-6 (PMC5854723; doi:10.1007/s00438-017-1401-6)
Supplement: Supplementary file 1 — Supplementary material 1 (PDF 1724 KB) [file 438_2017_1401_MOESM1_ESM.pdf]

Online Resources

Mapping causal mutations by exome sequencing in a wheat TILLING population: a tall mutant case study

Molecular Genetics and Genomics

Youngjun Mo, Tyson Howell, Hans Vasquez-Gross, Luis Alejandro de Haro, Jorge Dubcovsky, Stephen Pearce

Corresponding author:

Stephen Pearce, Department of Soil and Crop Sciences, Colorado State University, Fort Collins, Colorado, United States of America

stephen.pearce@colostate.edu

Fig. S1 *Rht-B1* amplification from the 48 M_4 mapping individuals. PCR was conducted using primers amplifying 929 bp to 2397 bp region of *Rht-B1* [21]. Target bands in the gel picture are indicated with grey triangles. Blue and red colors indicate the 24 shortest and 24 tallest M_4 lines, respectively. C, T, and H indicate homozygous C SNP, homozygous T SNP, and heterozygous CT genotypes, respectively, for the peak SNP (C3986T on IWGSC_CSS_4BS_scaffold_4881784; see Table 1 and Online Resource Table S2).

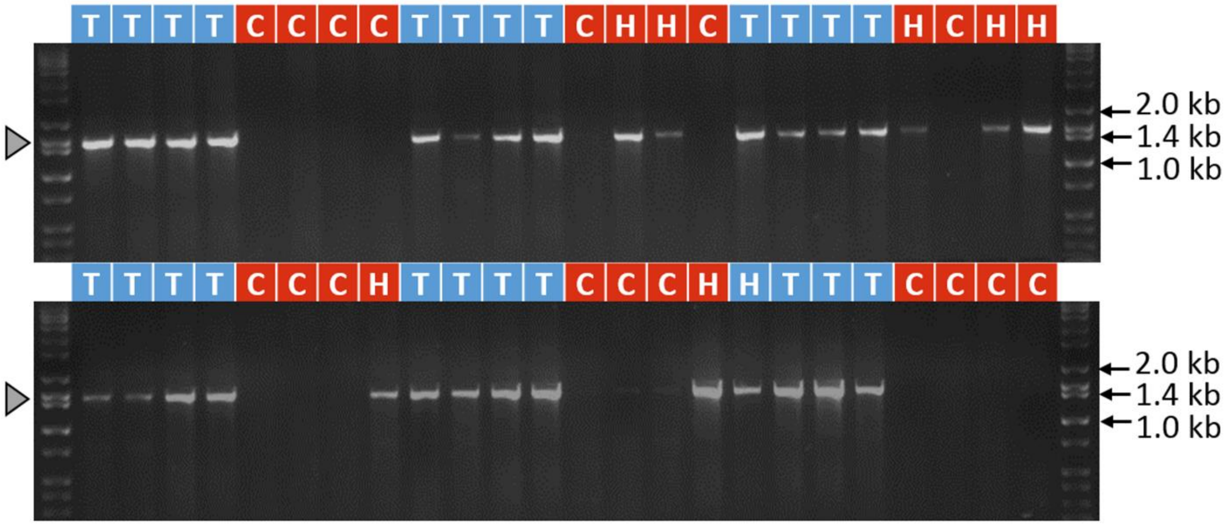

Fig. S2 Coverage analysis of genes residing in the deletion identified in T4-3822 and its flanking region. Nine genes in the deleted region (Table 2) and three flanking genes at either side of the deletion analyzed in detail from the coverage plot in Fig. 4a. For each gene, full coding region and 200 bp of 5’ and 3’ flanking sequences (according to the IWGSC RefSeq v1.0 annotation) were selected. Gene IDs and their locations on chromosome 4B are highlighted in bold to indicate their deletion in tall M_4 sister lines. Blue, purple, and red colors in coverage plots indicate M_4 individuals homozygous (CC), heterozygous (CT), and homozygous (TT) for the peak SNP (C3986T on IWGSC_CSS_4BS_scaff_4881784), respectively (Online Resource Table S2). X-axis: position on chromosome 4B (bp); Y-axis: normalized read depth.

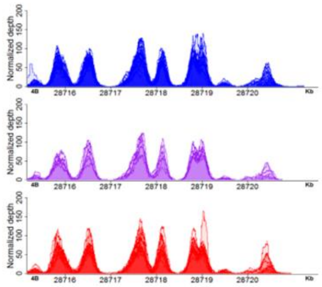

TraesCS4B01G042000 (28.72 Mb)

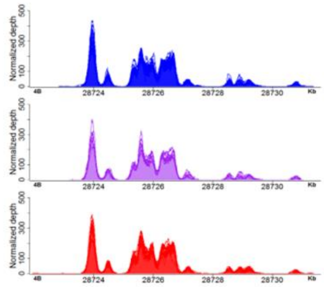

TraesCS4B01G042100 (28.72 Mb)

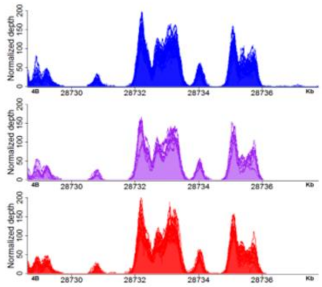

TraesCS4B01G042200 (28.73 Mb)

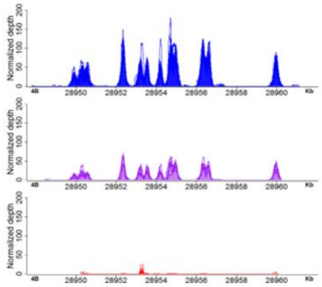

TraesCS4B01G042300 (28.95 Mb)

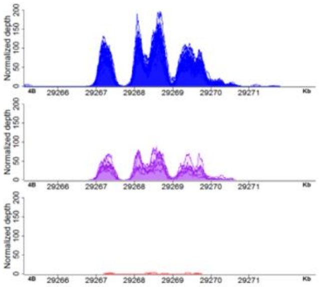

TraesCS4B01G042400 (29.27 Mb)

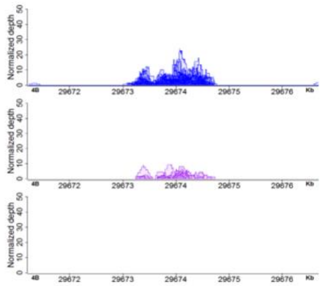

TraesCS4B01G042500 (29.67 Mb)

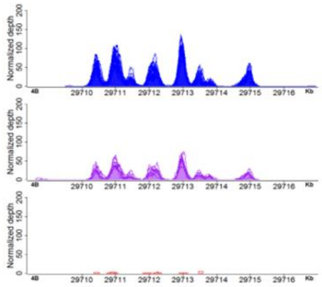

TraesCS4B01G042600 (29.71 Mb)

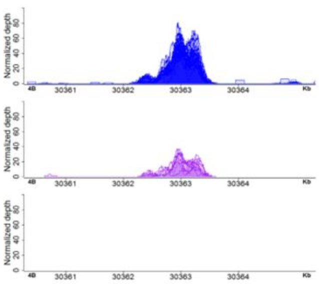

TraesCS4B01G042700 (30.36 Mb)

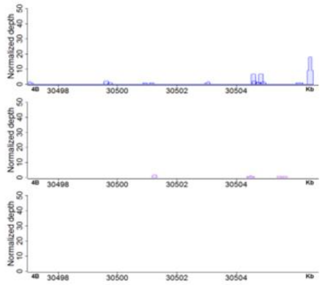

TraesCS4B01G042800 (30.50 Mb)

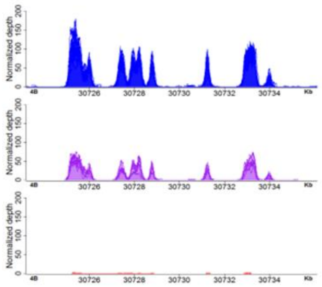

TraesCS4B01G042900 (30.73 Mb)

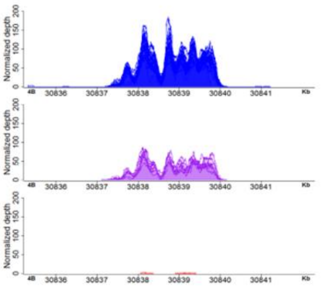

TraesCS4B01G043000 (30.84 Mb)

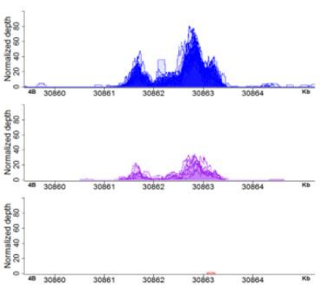

TraesCS4B01G043100 (30.86 Mb)

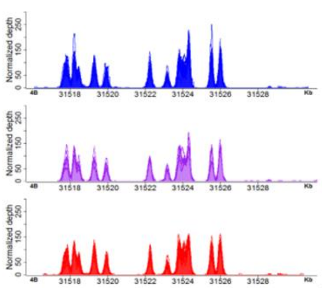

TraesCS4B01G043200 (31.52 Mb)

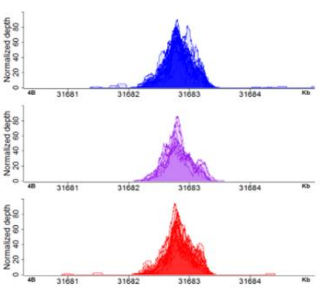

TraesCS4B01G043300 (31.68 Mb)

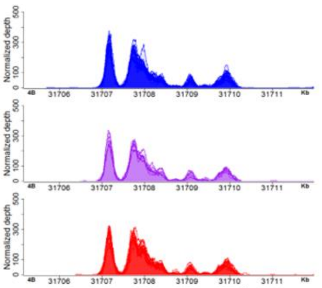

TraesCS4B01G043400 (31.71 Mb)

Fig. S3 Bulk size simulation. BSA rank (A) and |Δ(SNP-index)| (B) of the three most significant SNPs (Table 1) at different bulk sizes. BSA rank was determined by the |Δ(SNP-index)| values using modified competition ranking (whereby tied SNPs share the lowest rank).

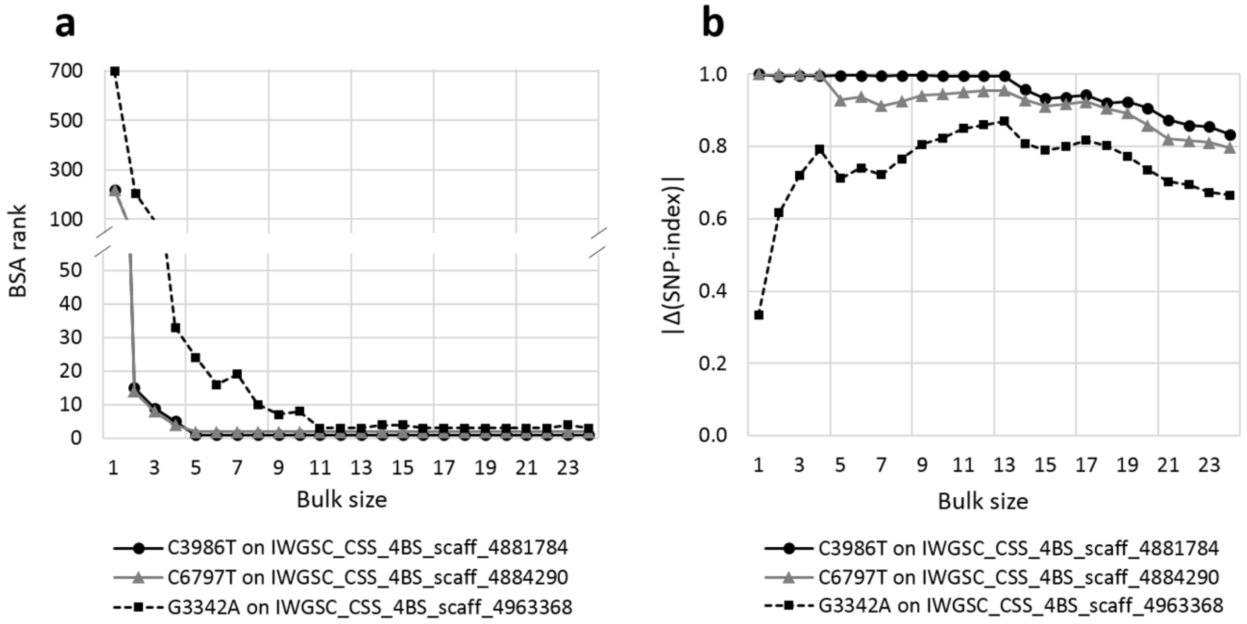

Fig. S4 BSA simulation with different bulk sizes and sequencing depths. (A) BSA simulation under six different sequencing depths when n = 24 (the 24 shortest and 24 tallest lines for each bulk). (B) BSA simulation under six different sequencing depths when n = 12 (the 12 shortest and 12 tallest lines for each bulk).

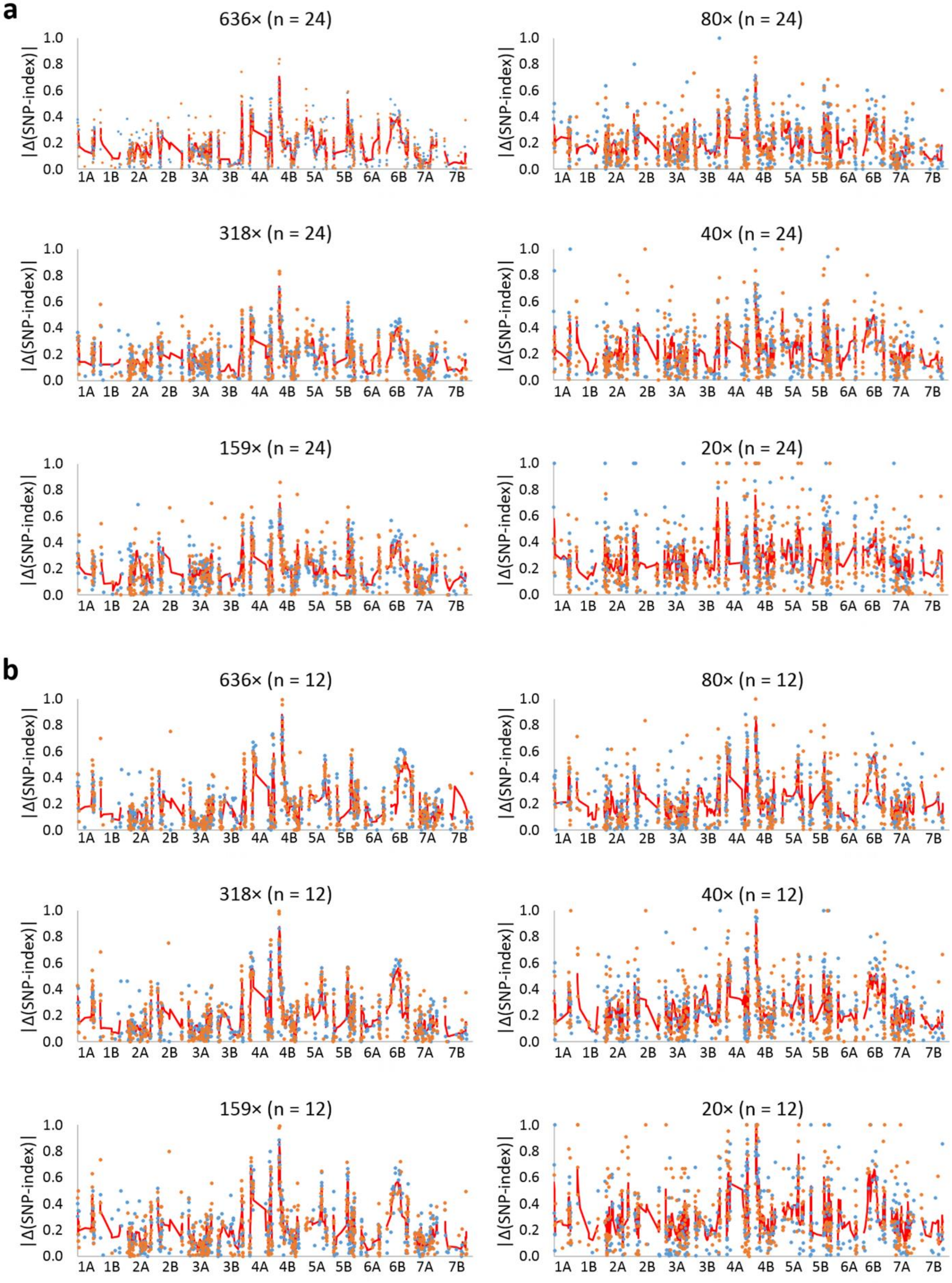
a
636× (n = 24)
80× (n = 24)
318× (n = 24)
40× (n = 24)
159× (n = 24)
20× (n = 24)
b
636× (n = 12)
80× (n = 12)
318× (n = 12)
40× (n = 12)
159× (n = 12)
20× (n = 12)
|Δ(SNP-index)|
0.0 0.2 0.4 0.6 0.8 1.0
1A 1B 2A 2B 3A 3B 4A 4B 5A 5B 6A 6B 7A 7B

Fig S5 Effect of the *Rht-B1* W424* mutation on plant height in an M_4 population (n = 16) derived from TILLING line T4-3545. M_4 seeds bulk-harvested from an M_3 row were used to evaluate plant height under greenhouse condition.

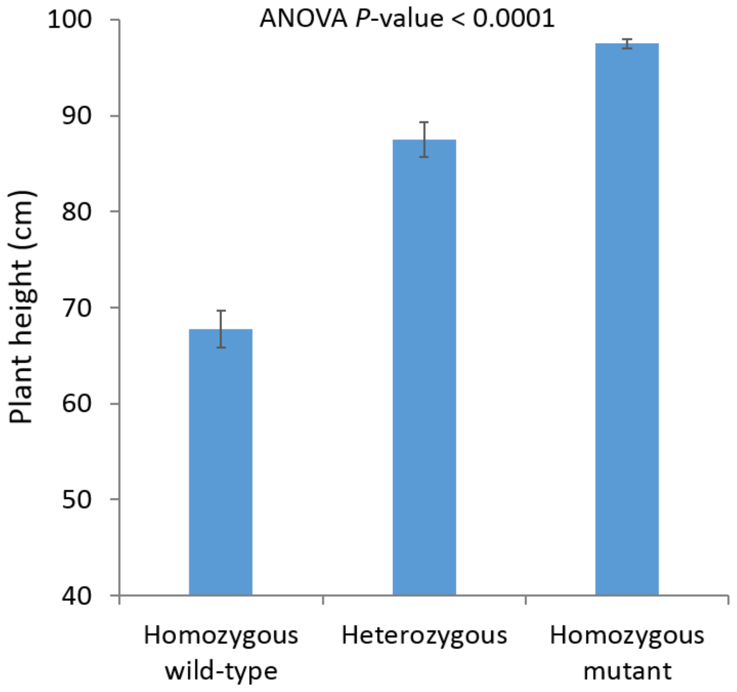

Fig S6 GA sensitivity assay. Coleoptile length (A) and shoot length (B) of 10-day old seedlings of wild-type Kronos (*Rht-B1b*, GA-insensitive), Gredho (*Rht-B1a*, GA-sensitive) and an M_5 line of T4-3822 homozygous for the deletion on chromosome 4BS treated with different concentrations of GA_3. *P* values are from one-way ANOVAs with four GA levels within each genotype. An asterisk indicates significant difference compared to the mock treatment within each genotype by Dunnett's test ($P < 0.05$).

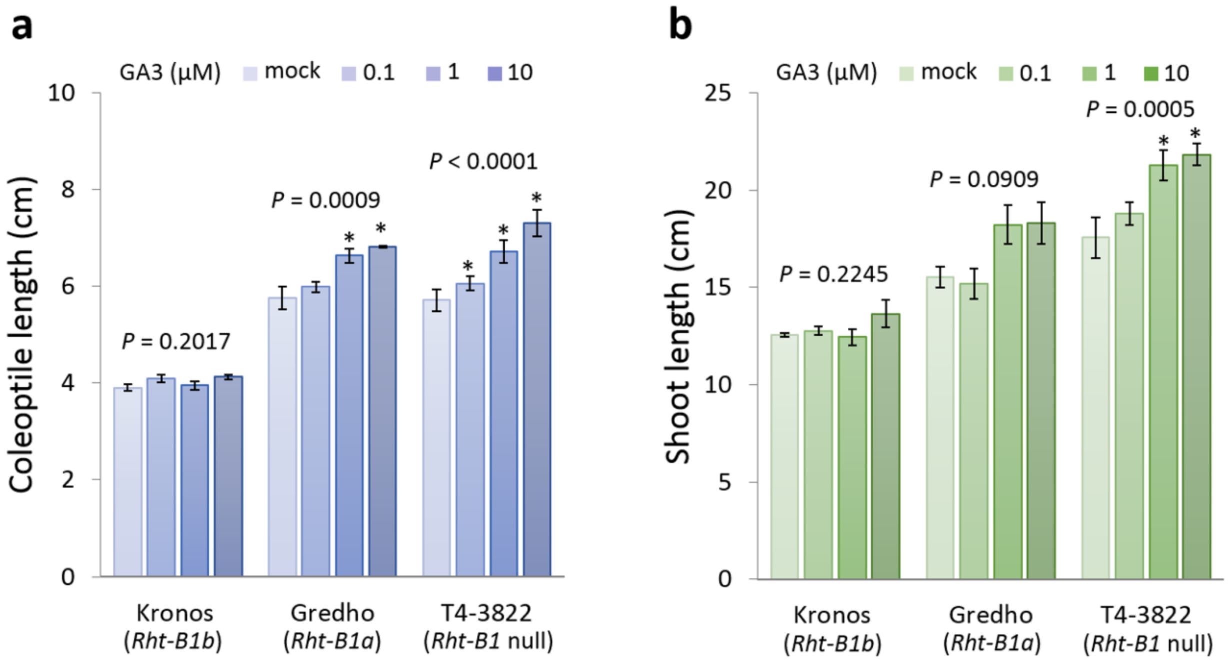

Table S1 Exome sequencing data summary of the 48 M_4 mapping individuals.

Plant	Number of raw reads	Number of trimmed reads	Number of mapped reads	Percentage of reads mapped	Number of uniquely mapped reads (quality >20)	Percentage of reads uniquely mapped (quality >20)
Plant 01	32,850,160	32,656,660	31,889,527	97.7	28,543,689	87.4
Plant 02	57,354,648	56,955,498	55,610,777	97.6	49,817,194	87.5
Plant 03	24,968,448	24,821,016	24,228,738	97.6	21,675,040	87.3
Plant 04	52,905,554	52,578,904	51,308,462	97.6	45,900,805	87.3
Plant 05	14,951,350	14,870,598	14,101,901	94.8	11,554,433	77.7
Plant 06	58,409,068	58,056,552	56,641,621	97.6	50,638,645	87.2
Plant 07	56,083,916	55,741,116	54,390,360	97.6	48,699,610	87.4
Plant 08	60,085,722	59,695,162	58,294,619	97.7	52,250,663	87.5
Plant 09	45,111,640	44,872,426	43,472,552	96.9	38,469,572	85.7
Plant 10	62,499,738	62,126,602	60,243,400	97.0	53,465,983	86.1
Plant 11	63,100,394	62,699,330	60,838,936	97.0	54,045,838	86.2
Plant 12	57,041,284	56,694,350	55,019,134	97.0	48,866,367	86.2
Plant 13	27,096,672	26,939,332	26,149,840	97.1	23,236,418	86.3
Plant 14	75,106,796	74,641,980	72,245,992	96.8	64,057,268	85.8
Plant 15	30,418,658	30,222,266	29,369,964	97.2	26,148,457	86.5
Plant 16	21,579,282	21,428,248	20,816,903	97.1	18,501,818	86.3
Plant 17	46,766,842	46,348,364	45,177,329	97.5	40,383,560	87.1
Plant 18	39,648,674	39,306,890	38,240,225	97.3	34,050,732	86.6
Plant 19	38,133,756	37,778,292	36,711,100	97.2	32,617,102	86.3
Plant 20	40,843,828	40,509,524	39,460,170	97.4	35,224,368	87.0
Plant 21	56,528,836	56,000,856	54,504,488	97.3	48,652,086	86.9
Plant 22	36,166,678	35,869,682	34,885,931	97.3	31,077,320	86.6
Plant 23	43,060,118	42,665,994	41,568,737	97.4	37,145,146	87.1
Plant 24	37,941,910	37,611,210	36,659,767	97.5	32,764,926	87.1
Plant 25	45,510,206	45,130,224	43,463,138	96.3	37,710,136	83.6
Plant 26	53,124,840	52,667,874	50,645,326	96.2	43,833,498	83.2
Plant 27	19,769,548	19,606,654	18,875,556	96.3	16,354,706	83.4
Plant 28	53,371,552	52,947,652	50,918,774	96.2	44,100,033	83.3
Plant 29	49,197,668	48,759,356	46,966,862	96.3	40,779,173	83.6
Plant 30	49,016,796	48,568,738	46,754,828	96.3	40,523,856	83.4
Plant 31	50,701,356	50,251,738	48,437,283	96.4	42,079,540	83.7
Plant 32	46,964,250	46,524,290	44,878,631	96.5	39,003,478	83.8
Plant 33	56,083,868	54,988,438	53,455,791	97.2	47,525,437	86.4
Plant 34	75,316,226	73,720,298	71,788,542	97.4	63,998,365	86.8
Plant 35	62,141,290	60,867,158	59,184,498	97.2	52,630,540	86.5
Plant 36	7,356,736	7,209,608	7,017,934	97.3	6,256,537	86.8
Plant 37	32,034,446	31,347,440	30,527,634	97.4	27,229,153	86.9
Plant 38	32,041,662	31,223,544	30,442,254	97.5	27,217,817	87.2
Plant 39	27,315,966	26,748,922	26,036,465	97.3	23,213,213	86.8
Plant 40	26,268,936	25,769,662	25,086,615	97.3	22,341,293	86.7
Plant 41	35,713,538	34,797,262	33,877,005	97.4	30,140,368	86.6
Plant 42	54,355,402	52,896,440	51,312,020	97.0	45,320,264	85.7
Plant 43	83,540,556	81,708,360	79,392,869	97.2	70,590,589	86.4
Plant 44	13,091,930	12,809,478	12,451,996	97.2	11,077,172	86.5
Plant 45	41,590,416	40,584,256	39,481,050	97.3	35,193,279	86.7
Plant 46	29,909,708	29,247,960	28,469,166	97.3	25,389,333	86.8

Plant 47	34,775,974	34,036,868	33,138,148	97.4	29,554,630	86.8
Plant 48	34,133,626	33,350,068	32,479,190	97.4	29,006,641	87.0
**Average**	**43,582,926**	**43,059,440**	**41,810,668**	**97.1**	**37,059,502**	**86.1**

Table S2 Plant height and genotype data of the significant SNPs (Bonferroni $P < 0.05$) on chromosome 4BS of the 48 M_4 mapping individuals.

Height (cm)[a]	IWGSC_CSS_4BS_scaff_	4920499	4933555	1070968	4872575	4909391	4961119	4963368	4884290	4881784	4911525	2307753	4868930	4904809	4935065
	SNP position	9117	4037	1979	19299	8166	20843	3342	6797	3986	11750	3238	6350	9422	7502
	Wile-type allele	G	G	C	C	A	G	G	C	C	C	C	C	G	C
	Mutant allele	A	A	T	T	G	A	A	T	T	T	T	T	A	T
	P-value	5.8E-06	8.8E-07	8.8E-07	7.4E-07	3.6E-06	1.7E-06	1.6E-11	1.1E-14	< 2.0E-16	4.8E-06	2.4E-06	2.7E-07	3.3E-05	1.1E-05
70	Plant 02	AA	GG	CC	CC	AA	AA	GG	TT	TT	TT	CC	CC	GG	CC
71	Plant 28	AA	GG	CC	CC	AA	AA	GG	TT	TT	TT	CC	CC	GG	CC
74	Plant 33	AA	GG	CC	CC	AA	AA	GG	TT	TT	TT	CC	CC	HET	HET
76	Plant 44	AA	GG	CC	CC	AA	AA	GG	TT	TT	.	.	CC	HET	HET
79	Plant 25	AA	GG	CC	CC	AA	AA	GG	TT	TT	CC	TT	TT	AA	TT
80	Plant 19	AA	GG	CC	CC	AA	AA	GG	TT	TT	TT	CC	CC	HET	HET
80	Plant 36	AA	GG	CC	CC	.	AA	GG	.	TT	.	.	HET	.	HET
82	Plant 01	AA	GG	CC	CC	AA	AA	GG	TT	TT	HET	.	HET	AA	TT
82	Plant 11	AA	GG	CC	CC	AA	AA	GG	TT	TT	CC	TT	TT	AA	TT
82	Plant 20	AA	GG	CC	CC	AA	AA	GG	TT	TT	CC	TT	TT	AA	TT
83	Plant 43	AA	GG	CC	CC	AA	AA	GG	TT	TT	TT	HET	HET	HET	HET
83	Plant 35	HET	GG	CC	CC	AA	AA	GG	TT	TT	TT	CC	CC	GG	CC
83	Plant 09	AA	GG	CC	CC	AA	AA	GG	TT	TT	TT	CC	CC	HET	HET
84	Plant 03	AA	GG	CC	CC	AA	AA	GG	TT	TT	TT	CC	CC	HET	HET
84	Plant 41	AA	GG	CC	CC	AA	AA	GG	TT	TT	.	TT	TT	AA	TT
84	Plant 10	AA	GG	CC	CC	AA	AA	GG	TT	TT	TT	CC	CC	GG	CC
84	Plant 04	AA	GG	CC	CC	AA	AA	GG	TT	TT	HET	HET	HET	HET	HET
84	Plant 27	HET	HET	HET	HET	HET	HET	HET	HET	HET	HET	HET	HET	HET	HET
85	Plant 17	AA	GG	CC	CC	AA	AA	GG	TT	TT	TT	CC	CC	GG	CC
85	Plant 18	AA	GG	CC	CC	AA	AA	HET	HET	TT	TT	CC	CC	GG	CC
85	Plant 42	AA	GG	CC	CC	AA	AA	GG	TT	TT	CC	TT	TT	AA	TT
85	Plant 26	AA	GG	CC	CC	AA	AA	GG	TT	TT	TT	CC	CC	GG	CC
86	Plant 12	AA	GG	CC	CC	AA	AA	GG	TT	TT	TT	CC	CC	HET	HET
86	Plant 34	AA	GG	CC	CC	AA	AA	GG	TT	TT	.	HET	HET	HET	HET
109	Plant 21	AA	GG	CC	CC	AA	AA	HET	HET	HET	HET	HET	HET	HET	HET
109	Plant 40	AA	GG	CC	CC	AA	AA	GG	TT	HET	.	HET	.	HET	HET
110	Plant 23	HET	HET	HET	HET	HET	HET	HET	HET	HET	HET	HET	HET	AA	TT
111	Plant 14	HET	HET	HET	HET	HET	HET	HET	HET	HET	HET	HET	HET	AA	TT
111	Plant 24	HET	HET	HET	HET	.	HET	HET	HET	HET	TT	HET	HET	AA	TT
114	Plant 37	AA	GG	CC	CC	AA	AA	GG	TT	CC	.	TT	TT	AA	TT

117	Plant 15	AA	GG	CC	CC	AA	AA	HET	HET	HET	HET	HET	.	HET	HET
117	Plant 08	GG	AA	TT	TT	GG	GG	AA	CC	CC	CC	TT	TT	AA	TT
118	Plant 46	AA	GG	CC	CC	AA	AA	AA	CC	CC	CC	TT	TT	AA	TT
119	Plant 05	GG	AA	TT	TT	GG	GG	.	CC	CC	.	.	TT	.	TT
120	Plant 32	HET	HET	HET	TT	GG	GG	AA	CC	CC	CC	TT	TT	AA	TT
120	Plant 16	HET	HET	HET	HET	.	.	HET	HET	HET	HET	HET	.	AA	TT
120	Plant 45	GG	AA	TT	TT	GG	GG	AA	CC	CC	CC	TT	TT	AA	TT
120	Plant 06	GG	AA	TT	TT	GG	GG	AA	CC	CC	CC	TT	TT	AA	TT
121	Plant 22	GG	AA	TT	TT	GG	GG	AA	CC	CC	CC	TT	TT	AA	TT
121	Plant 29	GG	AA	TT	TT	GG	GG	AA	CC	CC	CC	TT	TT	AA	TT
121	Plant 31	GG	AA	TT	TT	GG	GG	AA	CC	CC	CC	TT	TT	AA	TT
121	Plant 39	AA	GG	CC	CC	AA	AA	HET	HET	CC	CC	TT	TT	AA	TT
122	Plant 13	GG	AA	TT	TT	GG	GG	AA	CC	CC	CC	TT	TT	AA	TT
122	Plant 47	AA	GG	CC	CC	AA	AA	GG	HET	CC	CC	TT	TT	AA	TT
123	Plant 30	GG	AA	TT	TT	GG	GG	AA	CC	CC	CC	TT	TT	AA	TT
123	Plant 48	GG	AA	TT	TT	GG	GG	AA	CC	CC	CC	TT	TT	AA	TT
125	Plant 38	AA	GG	CC	CC	AA	AA	AA	CC	CC	CC	TT	TT	HET	HET
129	Plant 07	.	HET	HET	HET	HET	.	HET	CC	CC	CC	TT	TT	AA	TT

a – Plants shaded in blue = short phenotype; plants shaded in pink = tall phenotype.

Table S3 Primer sequences used to amplify the *Rht-B1* gene.

Rht-B1 region[a]	Primer name[b]	Sequence (5' to 3')[b]
-1888 to -648	Rht-B-F4	CACCACCGATCTCGAACAA
	Rht-ABD-R10	CATAGCTAATGCGACACACG
-986 to -177	Rht-B-F5	CAATGACAAAATAATAGCCATTCTC
	Rht-B-R3	AGCGGCAGCGTAGTAGTTGT
-449 to 84	Rht-F03	GAGTCTGACGCAGCAGAGAG
	Rht-ABD-R5	CGACACCATCATCTTGTCCT
-58 to 1047	Rht-B-F1	AGGCAAGCAAAAGCTTGAGA
	Rht-ABD-R6	TGCATCCCCTGCTTGATG
925 to 2397	Rht-ABD-F3	TTCTACGAGTCCTGCCCCTA
	Rht-B-R1	CGGCAAAGGAAGCTAAGTTG

[a]Position from the start codon. [b]Primer name and sequences are from Wilhelm et al. (2013).

Table S4 Nonsense and splice site mutations within the nine genes located in the 28.95 – 30.86 Mb deletion on chromosome 4B identified from the *in silico* wheat TILLING database.

Gene ID (RefSeq v1.0)	Gene ID (PGSB/MIPS version 2.2)	Nonsense mutation		Splice site mutation	
		Effect	TILLING line	Effect	TILLING line
TraesCS4B01G042300	Traes_4BS_940966B84	Q59*	T6-1044	C2660T (donor)	T4-2987
		Q125*	T6-1290	C2870T (donor)	T4-4307
		W221*	T4-3224, T6-1354	C5259T (donor)	T6-1532
		W293*	T6-0849	C5360A (acceptor)	T6-1553
		W330*	T4-1290		
		Q385*	T4-413		
		W427*	T6-0281		
TraesCS4B01G042400	Traes_4BS_00E6DD0F9	Q334*	T4-413	C1367T (donor)	T6-1103
TraesCS4B01G042500	Traes_4BS_3D1AEE34B	-	-	-	-
TraesCS4B01G042600	Traes_4BS_1706C4B88	W53*	T4-2587, T6-0926		
TraesCS4B01G042700	Traes_4BS_DC076554D	Q18*	T6-1759		
		Q142*	T4-2226		
		E306*	T6-1331		
		W341*	T4-562		
TraesCS4B01G042800	-	-	-	-	-
TraesCS4B01G042900	Traes_4BS_5D41A789A	W52*	T4-199	C5903T (acceptor)	T4-4657
		W158*	T4-2206		
		W186*	T4-3233		
		W187*	T6-0667		
		W233*	T4-2087, T6-1553		
TraesCS4B01G043000	Traes_4BS_668A7A78A	W350*	T6-0173	C2575T (acceptor)	T4-2166
				C2666T (donor)	T4-391
				C1996T (acceptor)	T6-2092
TraesCS4B01G043100 (*Rht-B1*)	Traes_4BS_2EE4988CD	Q291*	T6-1461		
		W424*	T4-3545		
		W435*	T6-1509		

[a] IWGSC RefSeq v1.0 annotation; [b] PGSB/MIPS version 2.2 annotation used for the sequenced TILLING database (Krasileva et al. 2017; www.wheat-tillin.com); [c] T4 and T6 lines derived from the tetraploid and hexaploid wheat varieties Kronos and Cadenza, respectively.

Text S1 False positive estimation in BSA simulation

The threshold of |Δ(SNP-index)| ≥0.8 was used to estimate the number of false positives in BSA simulation with different sequencing depths and bulk sizes (Fig 5B). The probability of having |Δ(SNP-index)| ≥0.8 at an unlinked SNP is 0.001% at sequencing depth n = 10, which is calculated using the following rationale.

Expected |ΔSNP-index| at the causal SNP (assuming phenotype accuracy):

SNP-index (W-bulk) = 0; SNP-index (M-bulk) = 1

|ΔSNP-index|= |1 – 0| = 1

Expected |ΔSNP-index| at a perfectly linked SNP (assuming phenotype accuracy):

1) Coupling phase

SNP-index (W-bulk) = 0; SNP-index (M-bulk) = 1

|ΔSNP-index|= |1 – 0| = 1

2) Repulsion phase

SNP-index (W-bulk) = 1; SNP-index (M-bulk) = 0

|ΔSNP-index|= |0 – 1| = 1

Expected |ΔSNP-index| at a non-causal, unlinked SNP (assuming phenotype accuracy):

SNP-index (W-bulk) = SNP-index (M-bulk) = 0.5

|ΔSNP-index|= |0.5 – 0.5| = 0

Probability of a non-causal, unlinked SNP with |ΔSNP-index| ≥ 0.8

= [Probability of ΔSNP-index ≥0.8] + [Probability of ΔSNP-index ≤-0.8]

For example, when the average sequencing depth in each bulk = 10,

1) Probability of ΔSNP-index ≥0.8

There are 6 cases (3 + 2 + 1) when ΔSNP-index ≥0.8

Case	No. of reads mapping to the mutant allele		SNP-index		
	W-bulk	M-bulk	W-bulk	M-bulk	ΔSNP-index
1	0	10	0	1	1
2	1	10	0.1	1	0.9
3	2	10	0.2	1	0.8
4	0	9	0	0.9	0.9
5	1	9	0.1	0.9	0.8
6	0	8	0	0.8	0.8

Probability of ΔSNP-index ≥0.8 = (3 + 2 + 1) * (0.5^{10}) * (0.5^{10})

2) Probability of ΔSNP-index ≤-0.8

There are 6 cases (3 + 2 + 1) when ΔSNP-index ≤-0.8

Case	No. of reads mapping to the mutant allele		SNP-index		
	W-bulk	M-bulk	W-bulk	M-bulk	ΔSNP-index
1	10	0	1	0	-1
2	10	1	1	0.1	-0.9
3	10	2	1	0.2	-0.8
4	9	0	0.9	0	-0.9
5	9	1	0.9	0.1	-0.8
6	8	0	0.8	0	-0.8

Probability of ΔSNP-index ≤-0.8 = (3 + 2 + 1) * (0.5^{10}) * (0.5^{10})

3) Probability of |ΔSNP-index| ≥0.8

= 2 * (3 + 2 + 1) * (0.5^{10}) * (0.5^{10})

To generalize, when the average coverage in each bulk = n

1) Probability of ΔSNP-index ≥0.8

There are (Int (0.2n) + 1) * (Int (0.2n) + 2) / 2 cases when ΔSNP-index ≥0.8.

(Int indicates integer value)

Probability of ΔSNP-index ≥0.8

= (Int (0.2n) + 1) * (Int (0.2n) + 2) / 2 * (0.5^n) * (0.5^n)

2) Similarly, probability of ΔSNP-index ≤-0.8

= (Int (0.2n) + 1) * (Int (0.2n) + 2) / 2 * (0.5^n) * (0.5^n)

3) Probability of |ΔSNP-index| ≥0.8

= 2 * (Int (0.2n) + 1) * (Int (0.2n) + 2) / 2 * (0.5^n) * (0.5^n)

= (Int (0.2n) + 1) * (Int (0.2n) + 2) * 0.5^{2n}

Based on this equation, probability of |ΔSNP-index| ≥0.8 at a non-causal, unlinked SNP at different coverage is as follows:

Coverage	5	10	20	40	80

Probability	0.0059	1.1444E-05	2.7285E-11	7.4446E-23	2.0937E-46

The actual number of estimated false positives with the threshold of |ΔSNP-index| ≥0.8 (Fig. 5b) is higher than the calculated probability above. This is because the capture efficiency is highly variable and there are many low-depth regions even under high (~40×) average coverage. Therefore, as suggested in the discussion section of the manuscript, we recommend targeting higher coverage (~80×) when conducting mapping-by-exome-sequencing experiments compared to performing mapping-by-whole-genome sequencing (10-40×).
